# Leveraging mRNA cMH and machine learning for the deconvolution of mixed body fluid stains in East Asian populations

**DOI:** 10.1093/bib/bbag540

**Published:** 2026-10-19

**Authors:** Qinglin Liu, Xiaolian Wu, Fanzhang Lei, Xi Yuan, Meiming Cai, Yiman Wu, Qiong Lan, Chunmei Shen, Bofeng Zhu

**Affiliations:** Guangzhou Key Laboratory of Forensic Multi-Omics for Precision Identification, School of Forensic Medicine, Southern Medical University, 1023–1063 Shatai South Road, Baiyun District, Guangzhou 510515, Guangdong, China; Guangzhou Key Laboratory of Forensic Multi-Omics for Precision Identification, School of Forensic Medicine, Southern Medical University, 1023–1063 Shatai South Road, Baiyun District, Guangzhou 510515, Guangdong, China; Guangzhou Key Laboratory of Forensic Multi-Omics for Precision Identification, School of Forensic Medicine, Southern Medical University, 1023–1063 Shatai South Road, Baiyun District, Guangzhou 510515, Guangdong, China; Guangzhou Key Laboratory of Forensic Multi-Omics for Precision Identification, School of Forensic Medicine, Southern Medical University, 1023–1063 Shatai South Road, Baiyun District, Guangzhou 510515, Guangdong, China; Guangzhou Key Laboratory of Forensic Multi-Omics for Precision Identification, School of Forensic Medicine, Southern Medical University, 1023–1063 Shatai South Road, Baiyun District, Guangzhou 510515, Guangdong, China; Guangzhou Key Laboratory of Forensic Multi-Omics for Precision Identification, School of Forensic Medicine, Southern Medical University, 1023–1063 Shatai South Road, Baiyun District, Guangzhou 510515, Guangdong, China; Guangzhou Key Laboratory of Forensic Multi-Omics for Precision Identification, School of Forensic Medicine, Southern Medical University, 1023–1063 Shatai South Road, Baiyun District, Guangzhou 510515, Guangdong, China; Department of Laboratory Medicine, Nanfang Hospital, Southern Medical University, 1838 Guangzhou Avenue North, Baiyun District, Guangzhou 510515, Guangdong, China; Guangzhou Key Laboratory of Forensic Multi-Omics for Precision Identification, School of Forensic Medicine, Southern Medical University, 1023–1063 Shatai South Road, Baiyun District, Guangzhou 510515, Guangdong, China; Key Laboratory of Forensic Medicine in Shanxi Province, School of Forensic Medicine, Shanxi Medical University, 98 Daxue Street, Yuci District, Jinzhong 030600, Shanxi, China

**Keywords:** body fluid stain, coding-region microhaplotype (cMH), mixture deconvolution, donor attribution, machine learning

## Abstract

Body fluid identification methods have become increasingly well-established, but deconvoluting multi-component mixtures remains a significant challenge. When dealing with complex mixtures, methods based on mRNA coding-region SNPs (cSNPs) still face limitations such as limited polymorphisms of markers and insufficient population specificity. mRNA coding-region microhaplotypes (cMHs), combining tissue specificity with high discrimination power, offer a promising alternative for donor attribution in body fluid stains. In this study, we developed a targeted RNA sequencing panel for East Asian populations, comprising 36 body fluid-specific genes containing 48 cMHs (including 108 cSNPs), along with five genes lacking cMHs. This panel enables both tissue source identification and donor attribution for five types of body fluids and their mixtures. Through specificity evaluation of single-source body fluid stains, we identified six novel body fluid-specific genes. Furthermore, by integrating machine learning algorithms with an *in silico* mixing strategy, we developed a body fluid classifier that achieved accurate inference for both single-source fluids and complex mixtures. The panel's cumulative discrimination power across the five body fluid types ranged from 0.99568126 (peripheral blood) to 0.99999999 (vaginal secretion). In conclusion, the dual-purpose panel demonstrates robust performance in both tissue source identification and donor attribution for single-source body fluids and complex mixtures.

## Graphical Abstract

**Graphical Abstract:**
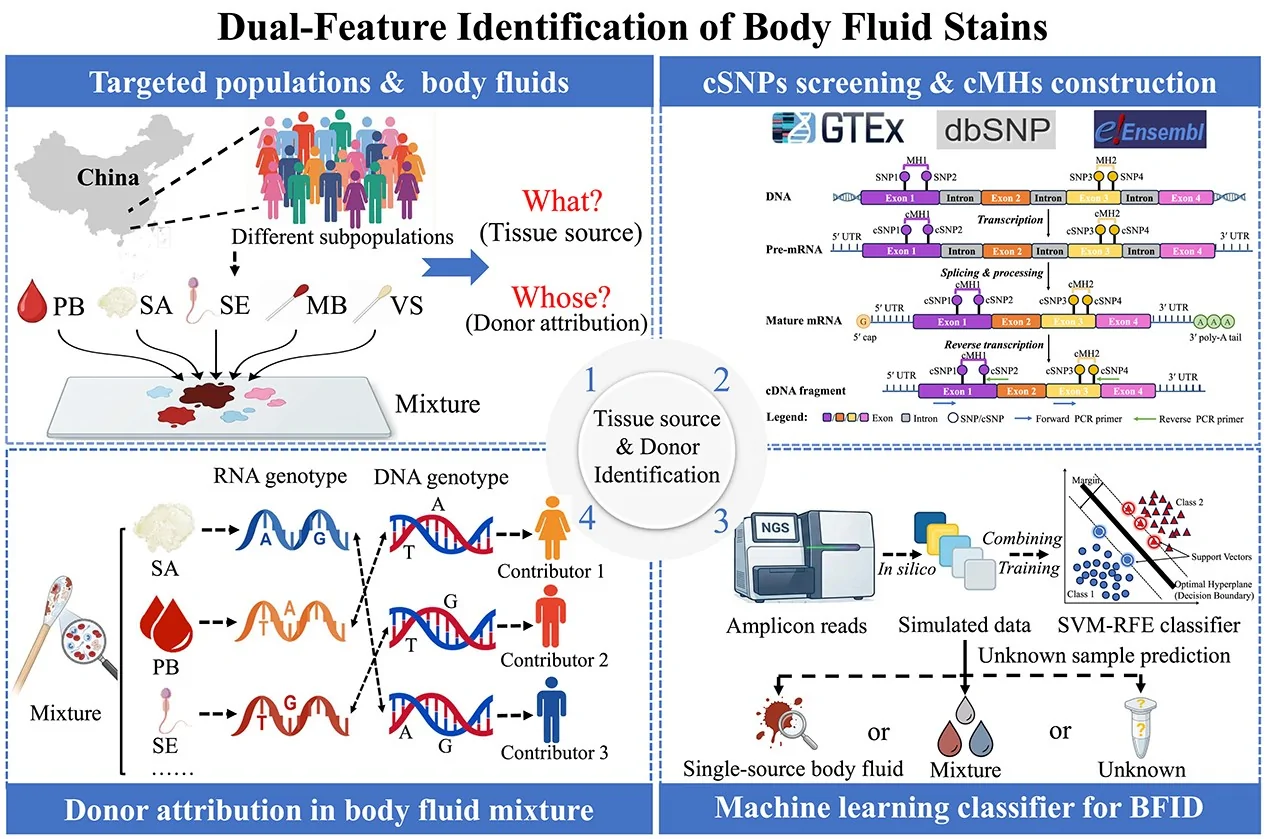

## Introduction

In criminal investigations, identifying the tissue source of biological stains, such as peripheral blood (PB), semen (SE), saliva (SA), vaginal secretion (VS), and menstrual blood (MB), is known as body fluid identification (BFID) and serves as a critical step in reconstructing crime scenes and characterizing the nature of the offense. Beyond BFID, accurately attributing these biological components to their respective donors (known as donor attribution) is equally vital, particularly in complex mixtures. In a well-documented case of sexual assault reported by Gill, a man was wrongfully accused due to the detection of his DNA in evidence collected at the scene. In reality, the DNA originated from the unintended transfer of his SA rather than SE. Gill termed this phenomenon the ‘association fallacy’, where a biological component is incorrectly attributed to the unintended donor. Under the hierarchical framework of forensic evidence evaluation, evidentiary value is assessed at different levels, including the source level (e.g., Mr. X is the donor of the blood) and the sub-source level (e.g., Mr. X is the donor of the DNA). Courts primarily focus on evidence evaluation at the source level. Crucially, the DNA Commission of the International Society for Forensic Genetics (ISFG) has provided guidelines for evidence evaluation related to different hierarchical propositions, explicitly stating that a likelihood ratio (LR) calculated at the sub-source level cannot be directly extrapolated to the source level [1]. Therefore, to avoid the association fallacy during donor attribution, the evidentiary value of STR profiling must not be directly extended to source-level hypotheses. To further evaluate source-level propositions, it is imperative that BFID methods provide additional information to directly attribute specific body fluid components to their respective donors.

To achieve precise donor attribution, forensic scholars have continuously sought to identify novel molecular markers and develop highly efficient detection assays. Harteveld et al. investigated the possibility of donor attribution based on the correlation between fluorescence signal intensities of DNA and RNA profiles on a capillary electrophoresis (CE) platform [2]. However, the findings indicated that, except for sex-associated fluids such as SE, VS, and MB samples, the DNA-to-RNA peak height ratios are influenced by multiple factors, including individual heterogeneity in gene expression and initial template amount. Subsequent research by Ingold et al. further supported this conclusion [3]. Another feasible approach utilizes DNA/RNA co-extraction technology to integrate mRNA analysis into the STR profiling workflow, enabling simultaneous BFID and donor attribution in a single multiplex PCR-CE assay [4, 5]. Unfortunately, constrained by limited multiplexing capacity, the efficacy of the method has not met expectations, and the risk of association fallacy remains. Compared with the CE platform, targeted RNA sequencing (RNA-seq) technology based on massively parallel sequencing (MPS) can simultaneously detect a larger number of mRNA markers, significantly enhancing the efficacy of mRNA profiling for BFID. Furthermore, this technology enables the detection of coding-region single nucleotide polymorphisms (cSNPs) on body fluid-specific mRNAs. Comparison of cSNP genotypes with an individual’s genomic DNA (gDNA) profile allows body fluid stains to be directly attributed to the corresponding donors, thereby avoiding the association fallacy [3, 6–9]. In a proof-of-concept study using the samples from a European population, Ingold et al. demonstrated the feasibility of the approach for donor attribution by detecting 35 cSNPs in 18 body fluid-specific mRNAs [3]. The method was further validated in the second EUROFORGEN/EDNAP collaborative exercise [6], and the researchers subsequently optimized the detection assay to develop an enhanced "BFID-cSNP-6F" panel containing 46 cSNPs from 23 body fluid-specific mRNAs. For a Caucasian population, the panel achieved discrimination power (DP) values ranging from 0.9570 to 0.9970 across five body fluids and skin tissue [7]. Several studies have developed various systems capable of simultaneous BFID and donor attribution analyses (dual-purpose) [10, 11]. However, some of these systems contain a limited number of cSNPs, restricting their efficacy in donor attribution. Furthermore, the selection of cSNPs in most current systems relies on allele frequency data from the five continental populations in the 1000 Genomes Project Phase 3 database (1KGP Phase 3). Due to the pronounced allele frequency heterogeneity among continental populations, those systems developed based on global data often exhibit substantial deviation between actual discriminatory power and theoretical expectation when applied to specific regional populations, leading to a decline in practical utility. Therefore, to enhance the efficacy of donor attribution, there is a pressing need to develop a dual-purpose panel that is tailored to specific regional populations and incorporates a greater number of highly polymorphic molecular markers.

Originally proposed by the Kidd laboratory, microhaplotype (MH) is defined as a genetic marker composed of two or more closely linked SNPs on a short DNA fragment (<300 bp) [12]. As a multi-allelic genetic marker, MH offers distinct advantages, such as the absence of stutter peak, low mutation rate, avoidance of preferential amplification of short fragment, and high polymorphism, making MH one of the most promising genetic markers in forensic practice. Numerous studies have demonstrated that MHs exhibit satisfactory potential for personal identification, mixture deconvolution, kinship testing, and biogeographical origin inference [13–16]. However, the application of MHs at the transcript level has not yet been fully explored, with no studies specifically investigating their potential application for donor attribution in body fluids. Consequently, we propose defining MHs consisting of two or more closely linked cSNPs within short segments (<250 bp) of mRNA coding regions as coding-region MHs (cMHs). The schematic structure of the cMH marker is illustrated in Fig. 1A, detailing the correspondence between mRNA molecules carrying cSNPs/cMHs and gDNA fragments containing SNPs/MHs. cMHs retain the forensic advantages of MHs, while exhibiting body fluid-specific expression profiles. Therefore, detecting cMH markers in body fluid-specific mRNAs offers a promising approach for both BFID and donor attribution.

**Figure 1:**
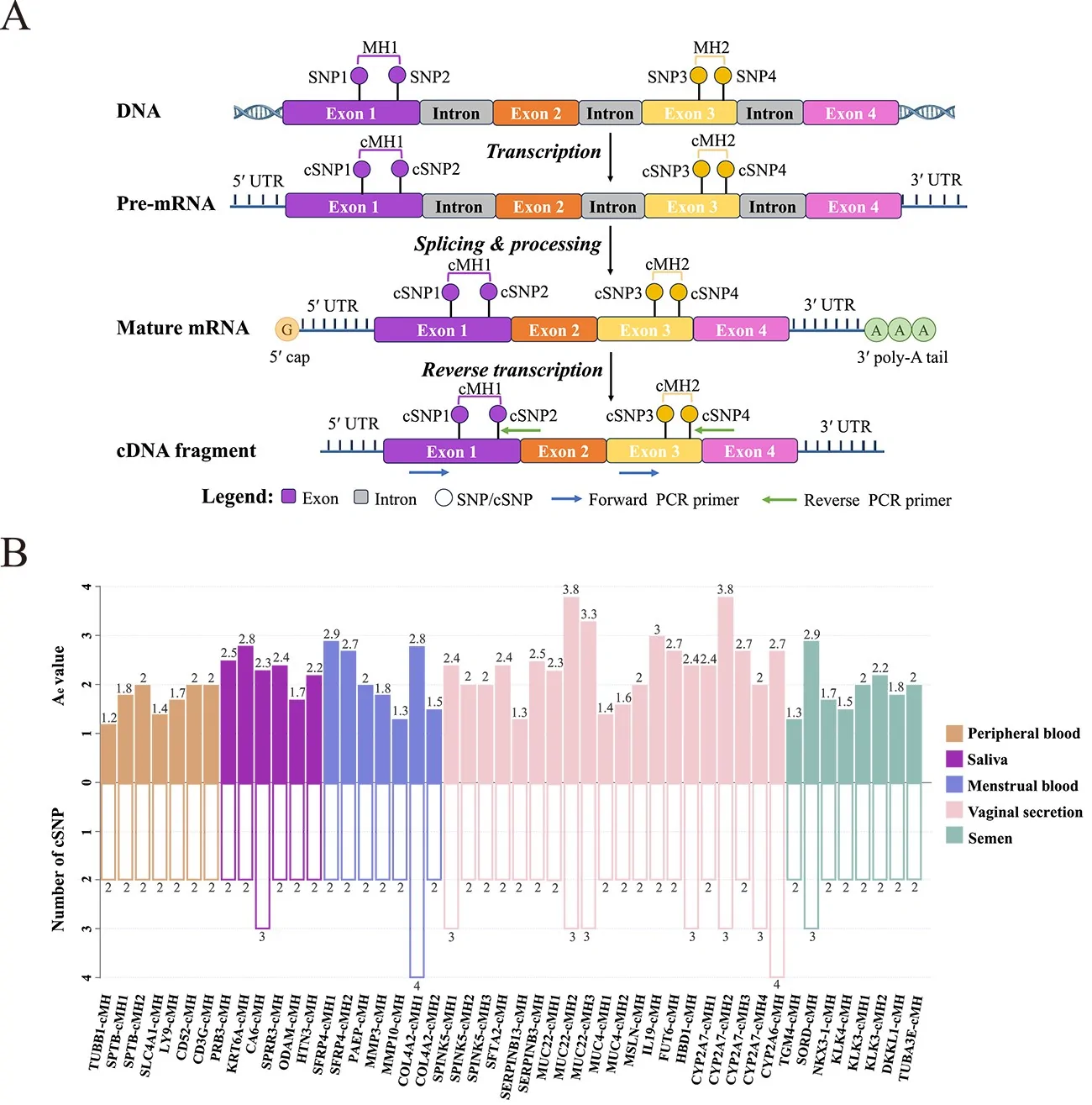
A. Schematic diagram of the structure and primer design for cMH markers. The figure illustrates the correspondence between cSNPs/cMHs on mRNA molecules and their homologous SNPs/MHs on genomic DNA fragments, and each cMH consists of two or more cSNPs located within the same exon. To ensure cDNA specificity, at least one PCR amplification primer is designed to span an exon-exon junction. B. Distribution of cSNP counts and effective number of alleles (*A_e_*) across the 48 cMHs.

In this proof-of-concept study, we developed a dual-purpose panel tailored for East Asian populations using targeted RNA-seq technology. The panel comprises 36 body fluid-specific genes containing 48 cMHs (encompassing 108 cSNPs), along with five genes lacking cMHs, and is designed to achieve BFID and donor attribution for five body fluid stains and their complex mixtures. The study thoroughly explored the potential applications of the panel in dual-feature identification of body fluid stains and provided a comprehensive and systematic evaluation of its sensitivity, specificity, stability, mixture deconvolution capability, and performance on challenging aged samples.

## Methods and materials

### Sample collection and body fluid stain preparation

Samples of PB, SE, MB, and VS (*n* = 25 each), and SA samples (*n* = 23), were collected from Chinese healthy volunteers aged 20–30 years. Prior to collection, all volunteers provided written informed consent. The collection protocol was reviewed and approved by the Ethics Committee of Southern Medical University (No. 2023–91), and the study was conducted in accordance with the principles of the Declaration of Helsinki as revised in 2024. Fresh liquid PB samples were collected in EDTA tubes. Liquid SA samples were collected in sterile, sealed plastic tubes after the volunteers had fasted for 1 h. SE samples were self-collected by the volunteers and immediately transported to the laboratory for processing. 200 μL aliquot of each of the aforementioned three body fluids was spotted onto sterile cotton swabs to prepare stains. For VS, volunteers performed self-collection using sterile cotton swabs by swabbing the vaginal wall one week after the completion of their menstrual cycle. MB samples were collected on the third day of the menstrual cycle using sterile cotton swabs. Volunteers were required to abstain from sexual intercourse for 3 days prior to the collection of SE and VS samples. All prepared swab samples were dried at room temperature for at least 12 h and subsequently stored at −80 °C.

To evaluate the sensitivity of the panel, sequencing was performed using five different total RNA input amounts (50 ng, 25 ng, 10 ng, 5 ng, and 1 ng), with one sample tested for each body fluid type at each input level. Additionally, the reproducibility of the assay was assessed using three technical replicates of each body fluid sample. Furthermore, to investigate the efficacy of the panel in mixture deconvolution, 40 complex mixtures were prepared using the samples from the aforementioned volunteers. For binary mixtures, two types were designed: mixtures with distinct donors and body fluids, and mixtures that originated from different donors but comprising the same body fluid type. These binary mixtures were formulated at typical ratios of 1:1 and 1:10, as well as highly unbalanced ratios of 1:20 and 1:30, to validate the panel's capacity to identify the minor component and its corresponding contributor accurately. All mixtures were prepared by blending total RNA extracts from the respective body fluids. To assess the panel's performance on degraded samples, challenging and aged samples were prepared with various substrates, storage durations, and specific environmental exposure conditions. All aged samples were maintained in a ventilated indoor environment. Detailed information regarding all mixtures and degraded samples is provided in Supplementary Note S1 available online at http://bib.oxfordjournals.org/.

### Selection of body fluid-specific cSNPs and cMH construction

Initially, previously reported body fluid-specific genes were screened as candidate genes from published literature [11, 17–30]. To further identify novel body fluid-specific genes, gene sets comprising the top 200 genes by expression level were retrieved from the GTEx Portal (https://www.gtexportal.org/home/) for five specific tissues: minor salivary gland, testis, uterus, vagina, and whole blood. Subsequently, differential expression analysis was performed using the DESeq2 package in *R*, with a threshold of log2 fold change (FC) > 5, to identify novel candidate genes showing significantly differential expression between target and non-target tissues. Following this, cSNPs that met the following selection criteria within the mRNA transcripts of all aforementioned candidate genes candidate genes were retrieved were retrieved from the dbSNP database to construct cMHs. The comprehensive workflow for cSNP screening and cMH construction is provided in Supplementary Note S2 available online at http://bib.oxfordjournals.org/. The specific selection criteria for cSNPs were as follows: (i) a minor allele frequency (MAF) ≥ 0.1 in East Asian populations from the 1KGP Phase 3 database [31], though this was lowered to 0.06 for a few selected genes lacking more suitable cSNPs; (ii) the presence of at least two adjacent cSNPs with a physical distance of less than 250 bp located on the same exon to facilitate cMH construction; (iii) cSNPs positioned within 250 bp upstream or downstream of exon-exon junctions, to restrict the amplicon size to <300 bp; and (iv) variant types restricted to coding-region, 3'-UTR, or 5'-UTR variants. Finally, the expression specificity of the novel candidate genes containing cMHs was further validated in five types of body fluid using reverse transcription quantitative PCR (RT-qPCR). This process resulted in the identification of new body fluid-specific genes, thereby enhancing the panel's efficacy. Notably, to further enhance the BFID efficacy of the panel, a few previously reported body fluid-specific genes lacking cMHs were also retained.

### Nucleic acid extraction, reverse transcription, and DNase I digestion

Total RNA was extracted from body fluid stains using RNAiso Plus (Takara Biomedical Technology, China). For RNA extraction from SE stains, the lysis buffer was supplemented with an additional 2 μL of 0.1 M DTT. Subsequently, total RNA concentrations were determined using the Qubit™ RNA HS Assay Kit (Invitrogen, USA) and a Qubit Fluorometer 4.0 (Invitrogen, USA). Prior to reverse transcription, 200 ng of total RNA in each reaction was subjected to gDNA digestion using Recombinant DNase I (Takara Biomedical Technology, China). To ensure complete digestion, the standard protocol was modified by reducing the reaction volume to 30 μL (from the recommended 50 μL) and extending the incubation time to 50 min (from the recommended 30 min). The efficacy of this modified gDNA digestion procedure was validated via qPCR assays using primers targeting intronic regions. Following gDNA removal, cDNA synthesis was performed using the TransScript® One-Step gDNA Removal and cDNA Synthesis SuperMix Kit (TransGen Biotech, China), using 71.5 ng of treated total RNA per 20 μL reaction (7 µL of the reaction, equivalent to 25 ng RNA input, was used for sequencing). During cDNA synthesis, no-template controls (NTCs) were included to monitor for exogenous contamination.

gDNA was extracted from various body fluid stains (PB, *n* = 8; SA, *n* = 8; MB, *n* = 9; VS, *n* = 8; SE, *n* = 7) using the MicroElute® Genomic DNA Kit (Omega Bio-tek, USA). The extracted gDNA was quantified using the Qubit™ dsDNA Quantification Assay Kit (Invitrogen, USA) and subsequently sequenced to evaluate genotype concordance between DNA and RNA profiling.

### Targeted amplification primer design

Two distinct primer pools were designed specifically for cDNA (primer pool 1) and gDNA (primer pool 2) templates, respectively. To ensure cDNA specificity and prevent gDNA co-amplification, at least one primer in each cDNA-targeting primer pair was required to span an exon-exon junction (as shown in Fig. 1A). As expected, this requirement was not applicable to primer pool 2. However, for primer pool 1, some primer pairs were confined to a single exon due to the inherent sequence constraints of certain transcripts. The design, synthesis, and specificity validation of all primers were performed by Sangon Biotech (Shanghai, China). To ensure relatively uniform coverage across all target regions, the primer pair mixing ratios were iteratively optimized to generate balanced read counts. Amplicon sizes for primer pool 1 ranged from 136 bp to 267 bp. Detailed information regarding the primer sequences for both pools is provided in Supplementary Table S1.

### Sequencing library preparation and data analysis

Except for sensitivity testing, the initial total RNA input for library preparation was 25 ng. Library construction for both RNA and DNA sequencing was performed using a two-step PCR method. The first round of amplification (30 μL) contained 7 μL of cDNA or gDNA template (for RNA and DNA libraries, respectively), 15 μL of 2× HiFi Master Mix, and 8 μL of the primer pool mixture. Following purification of the first-round amplification products according to the manufacturer's recommendations, they served as templates for the second-round PCR to incorporate index adapters. The PCR products were again purified using magnetic beads, rinsed with 80% ethanol, and thoroughly dried, after which the final DNA libraries were eluted in 20 μL of Elution Buffer. Subsequently, the DNA libraries were quantified using the Qubit™ dsDNA Assay Kit (Life Technologies, USA) to facilitate equimolar pooling. Finally, the pooled libraries were sequenced on the Illumina NovaSeq X Plus platform (Illumina, USA) in 150-bp paired-end (PE150) mode, following the manufacturer's instructions.

The raw sequencing data obtained underwent bioinformatics analysis through a systematic pipeline to achieve genotyping. First, stringent quality control was performed on the raw data: (i) Cutadapt (v1.2.1) was used to identify and trim sequencing adapters from both ends to prevent interference with subsequent alignment [32]; (ii) PRINSEQ-lite (v0.20.3) was employed for sequence quality trimming using a sliding window approach from the 3′ to 5′ end, removing bases with a quality score below 20 (i.e., an error rate exceeding 1%) [33]. The high-quality sequences retained after these steps were considered "clean reads" for downstream analysis. Subsequently, the clean reads were aligned to the reference sequences using the BWA-MEM algorithm of BWA (v0.7.13-r1126) with default parameters [34]. Reference sequences for the two primer pools were derived from mRNA and gDNA sequences retrieved from the NCBI Gene database (https://www.ncbi.nlm.nih.gov/gene/), respectively. Because the former comprises exclusively mature, intron-free mRNA sequences, these mRNA-derived reference sequences are suitable for alignment with the BWA-MEM algorithm. The SAM files obtained were sorted and converted to BAM format. Finally, variant calling was performed based on the alignment results using the mpileup command of SAMtools (v0.1.18) in conjunction with bcftools to generate VCF files for all target loci [35]. Using paired-end read information from the BAM files, we phased target cSNPs within the same amplicon using the ReadBackedPhasing module of GATK v3.8, and the results were integrated into the VCF files to determine the cMH genotypes.

Regarding the sequencing depth, a minimum amplicon coverage threshold was set at 500×, and amplicon with coverage below this threshold were converted to zero [17]. In addition, a minimum coverage threshold per sample was set at 5000×, and samples falling below this threshold were excluded from subsequent analyses. In previous studies, when the specific read counts of a body fluid type accounted for less than 1% of the total reads in a mixture, these reads were attributed to DNA contamination or cross-expression, and the corresponding body fluid component was thus deemed absent [7, 11]. However, background reads resulting from cross-expression exhibit significant heterogeneity, and their read counts are highly susceptible to sequencing depth. To address this, we calculated, for each body fluid type, the proportion of reads mapping to its corresponding body fluid-specific genes relative to the total reads in each non-target sample. The 95th percentile of this proportion distribution was subsequently established as the threshold to determine the presence of the corresponding body fluid component in the mixtures. Furthermore, principal component analysis (PCA) was conducted on all single-source data using *R* to assess sequencing batch effects, and a clustering plot was generated via the *ggplot2* package.

### Construction of body fluid classification models integrating machine learning algorithms and an *in silico* mixing strategy

Currently, classification scoring systems and data-driven models represent the two mainstream approaches for BFID. The former primarily encompasses the “x ≥ n/2” scoring system proposed by Lindenbergh et al., whereby a target body fluid is deemed present if at least 50% of its specific mRNA markers are detected in the assay, along with other optimized scoring methods derived from this framework [36]. Although these approaches are straightforward to implement, their performance still needs improvement. This limitation is particularly pronounced in highly unbalanced mixtures, where differentiating background reads caused by cross-expression from the low-proportion reads of minor components is challenging. The latter approach comprises predictive models constructed using various statistical, machine learning, or deep learning algorithms [37, 38]. While developing these models is generally more complex, they typically yield significantly higher analytical accuracy. In fact, machine learning algorithms are now widely applied in forensic science and bioinformatics fields, providing novel and robust solutions for deconvoluting complex scientific problems [39–41].

To preliminarily validate the BFID efficacy of the custom-developed panel, Random Forest (RF) and Support Vector Machine (SVM) multi-class models were constructed based on all amplicon reads to infer the tissue source of single-source body fluid stains. Prior to model training, the counts per million (CPM) values for each amplicon in the sequencing data after the previously described preprocessing steps were calculated, followed by a log2(CPM + 1) transformation for normalization. Additionally, Z-score standardization was applied exclusively to the input data for the SVM model. To strictly prevent data leakage, the 123 single-source body fluid samples were first partitioned into a training set and an independent test set in a 7:3 ratio, and the scaling parameters fitted exclusively on the training set were applied to transform the independent test set. During the training phase, a 10-fold cross-validation coupled with a grid search strategy was employed for hyperparameter optimization. Subsequently, the predictive performance of the models on the independent test set was evaluated using various metrics, including overall accuracy, confusion matrices, Macro F1-score, and the area under the receiver operating characteristic curve (AUROC). Finally, SHapley Additive exPlanations (SHAP) attribution analysis was incorporated to elucidate the decision-making logic of the model, thereby enhancing its transparency and interpretability.

Subsequently, to develop a universally applicable model also suitable for complex stains, such as mixtures and challenging specimens, five multi-label classification models were constructed using RF, SVM with Recursive Feature Elimination (SVM-RFE), eXtreme Gradient Boosting (XGBoost), Logistic Regression with Elastic Net penalty (Elastic Net LR), and k-Nearest Neighbor (KNN) algorithms to ultimately identify the optimal model. Initially, following the aforementioned dataset partitioning strategy, the single-source body fluid samples were divided into an initial training set (*n* = 86) and an independent test set (*n* = 37). To enhance the generalization capabilities of the models for complex mixtures, an *in silico* mixing strategy was applied to synthesize simulated mixture data based on amplicon read counts of the initial training set according to the following rules: (i) utilizing the 86 initial single-source samples, the algorithm exhaustively generated 2- to 5-component mixture expression profiles to ensure full coverage of all potential combinations; (ii) binary mixtures were configured at four mixing ratios (1:1, 1:10, 1:20, and 1:30), while multi-component mixtures were blended in equal proportions, generating 10 independent simulated expression profiles per combination; (iii) 5% Gaussian noise and a random feature dropout mechanism were introduced during data synthesis to simulate inter-individual variability in expression levels and marker loss caused by sample degradation. Ultimately, the 860 generated simulated mixture expression profiles were merged with the initial training set to form the final training set, which was used for 10-fold cross-validation training and grid-search hyperparameter optimization. During this process, the probability threshold that yielded the maximum F1-score, based on the precision-recall curve (PRC) from the 10-fold cross-validation, was selected as the optimal decision threshold for each body fluid component. Consequently, a specific body fluid component is deemed present only if its predictive probability exceeds the corresponding threshold. If all probabilities fall below their respective thresholds, the model outputs "Unknown". This strategy effectively mitigates the risk of forced classification for non-target biological samples. Additionally, the independent test set and an external validation set of 40 real mixtures (detailed in Supplementary Note S1 available online at http://bib.oxfordjournals.org/) served as unseen data to evaluate the models' generalization capacity using comprehensive metrics, such as the Exact Match Ratio (EMR), Hamming Loss, Macro F1-score, and Macro Area Under the ROC Curve (Macro AUC). In parallel, bootstrapping (1000 resamples) was employed to calculate the 95% confidence intervals (95% CIs) for each metric, systematically assessing model robustness. Notably, given the complex physiological compositions of MB, forensic prior knowledge (a masking procedure) was introduced during the models' performance evaluation to calibrate the prediction results, that is, to evaluate the prior knowledge-informed (hereafter referred to as prior-informed) classification performance. Specifically, when the model predicted the presence of PB, VS, or both within a MB-containing sample, the outcome was classified as a correct prediction via a masking process. Mechanistically, when the expression levels of PB- or VS-specific genes inherently expressed in a MB-containing sample exceed the predefined decision threshold, the multi-label classification model outputs the corresponding components based on objective signals. Therefore, rather than representing false positives, such predictions precisely reflect the endogenous compositional characteristics of MB. In other words, the prior-informed classification performance better reflects the model's ability to simultaneously identify both the actual components of the mixture and the biologically expected co-expressed components. Additionally, to objectively demonstrate the baseline classification capability of the models (i.e., without the masking procedure), the raw classification performance across both datasets was also reported and compared. This allows for an intuitive evaluation of the practical impact of forensic prior rules on the final predictions. Finally, McNemar's test was conducted on the detailed predictive results from the independent test and external validation sets to analyze the statistical significance of performance differences among models. The optimal model was thus identified by synthesizing these statistical findings with the aforementioned performance metrics. Thereafter, the statistical significance of the optimal model's feature learning was corroborated via a permutation test, and its anti-interference capacity and generalization performance were rigorously evaluated through model calibration analysis and the prediction of challenging samples. All model construction and evaluation procedures were executed in Python v3.13.3, using core open-source packages including *scikit-learn, xgboost*, and *shap*.

### Population genetic analysis

Genotype data for all cSNPs constituting the cMHs in East Asian populations were retrieved from the 1KGP Phase 3 database. Haplotype phasing was performed using PHASE v2.1 to infer the alleles and their respective frequencies for each cMH, subsequently allowing for the calculation of the effective number of alleles (*A_e_*) across all cMHs [42]. Pairwise linkage disequilibrium (LD) tests for all cMHs were conducted using the GENEPOP software [43]. Following this, cMHs exhibiting significant LD were merged into LD blocks, each treated as an independent single marker, and the forensic parameters for each marker were calculated using the STRAF v2.2.2 online tool (https://www.straf.fr/). To evaluate the overall discriminatory power of the panel in East Asian populations, the cumulative discrimination power (CDP) for each body fluid was calculated via the product rule. Furthermore, using genotype data for all cMHs in five continental populations (East Asia, South Asia, America, Europe, and Africa) in the 1KGP Phase 3 database, the fixation index (*F_ST_*) was calculated using *GENEPOP*, and PCA was performed to systematically assess population genetic difference.

## Results

### Development of a dual-purpose panel for body fluid stains in East Asian populations

#### Selection and validation of body fluid-specific genes and cMHs

Following the aforementioned cSNP selection criteria, 31 previously reported body fluid-specific genes containing cMHs were initially included. To ensure the identification efficacy of the panel for specific body fluids, five body fluid-specific genes (*ANK1, STATH, MMP11, PRM1*, and *SEMG1*) lacking cMHs were also retained exclusively for BFID. However, the preliminary sequencing results revealed that *KRT13*, a saliva-specific gene reported in previous literature, demonstrated high read counts in both PB and VS samples. Consequently, this gene was excluded from subsequent analyses. Ultimately, a total of 35 previously reported body fluid-specific genes were retained. Detailed functions of each gene are provided in Supplementary Table S2-1. Using RT-qPCR, we identified six body fluid-specific genes containing cMHs in transcripts retrieved from the GTEx Portal: *LY9* for PB, *CA6* and *ODAM* for SA, *COL4A2* for MB, and *DKKL1* and *TUBA3E* for SE (Supplementary Fig. S1). Thereafter, these novel body fluid-specific genes were incorporated into the targeted RNA-seq panel. Detailed information for the 41 genes is presented in Table 1.

**Table 1:** Detailed information of the 41 body fluid-specific genes and the number of corresponding cMHs.

Body fluid	Gene	Chromosome	Transcript	Number of cMHs
Peripheral blood	LY9^a^	1	NM_002348.4	1
	CD52	1	NM_001803.3	1
	ANK1^b^	8	NM_001142446.2	0
	CD3G	11	NM_000073.3	1
	SPTB	14	NM_001024858	2
	SLC4A1	17	NM_000342.4	1
	TUBB1	20	NM_030773.4	1
Saliva	SPRR3	1	NM_005416.3	1
	CA6^a^	1	NM_001215.4	1
	HTN3	4	NM_000200.3	1
	ODAM^a^	4	NM_017855.4	1
	STATH^b^	6	NM_003154.3	0
	PRB3	12	NM_006249.5	1
	KRT6A	12	NM_005554.4	1
Menstrual blood	SFRP4	7	NM_003014.4	2
	PAEP	9	NM_002571.4	1
	MMP10	11	NM_002425.3	1
	MMP3	11	NM_002422.5	1
	COL4A2^a^	13	NM_001846.4	2
	MMP11^b^	22	NM_005940.5	0
Vaginal secretion	IL19	1	NM_153758.5	1
	MUC4	3	NM_018406.7	2
	SPINK5	5	NM_001127698.2	3
	MUC22	6	NM_001318484.1	3
	SFTA2	6	NM_205854.3	1
	HBD1	8	NM_005218.4	1
	MSLN	16	NM_005823.6	1
	SERPINB13	18	NM_001307923.2	1
	SERPINB3	18	NM_006919.3	1
	CYP2A6	19	NM_000762.6	1
	CYP2A7	19	NM_000764.3	4
	FUT6	19	NM_001381957.1	1
Semen	TUBA3E^a^	2	NM_207312.3	1
	TGM4	3	NM_001648.2	1
	NKX3–1	8	NM_006167.4	1
	SORD	15	NM_003104.6	1
	PRM1^b^	16	NM_002761.3	0
	KLK3	19	NM_001648.2	2
	KLK4	19	NM_004917.5	1
	DKKL1^a^	19	NM_014419.4	1
	SEMG1^b^	20	NM_003007.5	0

a Body fluid-specific genes reported for the first time in this study.

b Genes lacking cMHs are exclusively for body fluid identification, not for donor attribution.

Initially, 51 candidate cMHs composed of 116 cSNPs were identified in the 36 body fluid-specific genes. During multiplex PCR design, three cMHs lacking suitable specific primers were excluded, resulting in a final dual-purpose panel comprising 48 cMHs (encompassing 108 cSNPs) in 36 genes, along with five genes lacking cMHs. The distributions of specific genes and their cMHs for each body fluid type in the panel were as follows: PB (7 genes/7 cMHs), SA (7 genes/6 cMHs), MB (6 genes/7 cMHs), VS (12 genes/20 cMHs), and SE (9 genes/8 cMHs). Most cMHs contained two cSNPs, whereas COL4A2-cMH1 and CYP2A6-cMH each contained up to four cSNPs. The *A_e_* is a metric that accounts for frequency bias, providing a more realistic reflection of the genetic diversity of a marker compared to the observed number of alleles [42]. In this study, the *A_e_* values of the 48 cMHs ranged from 1.3 to 3.8, with a mean of 2.2. Specifically, 32 cMHs exhibited *A_e_* values ≥2.0, indicating relatively high levels of polymorphism. Among all cMHs, TUBB1-cMH had the minimum *A_e_* value of 1.3, while MUC22-cMH2 and CYP2A7-cMH2 reached the maximum value of 3.8. The cSNP compositions and more details of the cMHs are provided in Supplementary Table S2-1, and the *A_e_* values for all cMHs are presented in Fig. 1B. Additionally, Supplementary Table S2-2 details the LD test results, revealing that 17 cMH pairs exhibited significant LD and were merged into seven LD blocks.

#### Genotype concordance between DNA and RNA profiling

To verify the reliability of targeted RNA-seq technology for genotyping, parallel sequencing was performed at both the RNA and DNA levels on 40 pairs of body fluid samples. To ensure genotyping accuracy, the analytical threshold (AT) for a single allele was set at 100×, meaning an allele was considered effectively detected only when its coverage exceeded this value. For heterozygote calling, the read count ratio of the minor allele to the major allele was required to be greater than 0.3. Furthermore, based on the methodology described by Ingold et al., the lower limit of detection for the minor allele was set at 0.1; specifically, when the aforementioned read count ratio fell between 0.1 and 0.3, it was defined as allelic imbalance. The results revealed that, for SPTB-cMH2, KRT6A-cMH, TUBA3E-cMH, and CYP2A7-cMH2, minor allele drop-out was observed in one or two targeted body fluid samples for each marker in the RNA profiling, resulting in pseudo-homozygous genotype calls. Notably, pseudo-homozygosity for TGM4-cMH was consistently observed in the RNA data of three SE samples, which aligns with the previous findings [3, 7, 11]. Furthermore, allelic imbalance was detected in CYP2A7-cMH2 and MUC22-cMH1 in one VS sample, and in MUC22-cMH3 in two VS samples. Supplementary Table S2-3 details the specific parameters (discordance frequency, RNA/DNA genotypes, and read proportions) of the aforementioned allelic imbalance or drop-out events. To investigate whether the occurrence of these phenomena was correlated with amplicon read counts, we further evaluated the read count distributions of these cMH markers in the paired RNA-seq data. As illustrated in Supplementary Fig. S2, allelic imbalance or drop-out events were not exclusively confined to amplicons with low reads, indicating that low sequencing depth is not the primary cause of these events in RNA-seq. Although the underlying mechanisms for these phenomena remain unclear, they may be attributed to random monoallelic expression or allelic expression imbalance [3, 44, 45]. Nevertheless, the RNA genotypes of the remaining cMHs were entirely consistent with their corresponding DNA genotypes, thereby demonstrating the reliability of cMH genotyping at the transcriptome level. Future studies should systematically investigate these discordant phenomena to decipher their underlying mechanisms and enhance the reliability of transcriptomic genotyping in forensic casework.

### Body fluid-specificity evaluation of the dual-purpose panel

The specificity of the panel, comprising 48 cMHs and five genes without cMHs, was evaluated using 123 single-source body fluid stains. The raw data obtained from the MPS platform were preprocessed according to the aforementioned criteria. The average read proportion per marker was calculated in all targeted body fluid samples, with the results illustrated in Fig. 2. In PB samples, the read counts of eight blood-specific markers accounted for 98.57% of the total reads (Fig. 2A). Minor expression of PAEP-cMH and CA6-cMH was also noted in some PB samples. These false-positive signals may be reduced by lowering the concentration of specific primers in the amplification system, as PAEP-cMH (average read count of 291 383) and CA6-cMH (average read count of 282 471) demonstrated extremely high read counts in their respective target body fluids. Additionally, weak cross-expression of SEMG1, SORD-cMH, and TUBA3E-cMH markers was observed in only a few PB samples, which is consistent with the findings from previous studies [17, 30]. For SA samples, seven SA-specific markers exhibited specific expression (Fig. 2B), contributing 97.93% of the total reads, with CA6-cMH accounting for the highest proportion at 43.19%. As expected, weak cross-expression of several VS-specific markers was observed in SA samples, contributing ~2.02% of the total reads. Previous research has suggested that this phenomenon may be attributed to the presence of epithelial cell components in both SA and VS [46]. In MB samples, the eight MB-specific markers accounted for 79.85% of the total reads, while VS- and PB-specific markers contributed 14.56% and 4.65%, respectively (Fig. 2C). This observation is primarily attributable to the unique physiological composition of MB, which is essentially a complex biological fluid composed of shed endometrial tissue, vaginal secretions, and blood. Furthermore, the fact that the panel includes more specific markers for VS than for MB partially contributed to the relatively high proportion of VS-specific marker reads in MB samples. Notably, low-level expression of a few SE- and SA-specific markers was also present in MB samples. Specifically, low read counts of SE-specific markers (SEMG1, SORD-cMH, and TUBA3E-cMH) and SA-specific markers (KRT6A-cMH and SPRR3-cMH) were detected in some MB samples. These results corroborate the previous reports regarding false-positive interference of these genes in MB samples [3, 17, 30].

**Figure 2:**
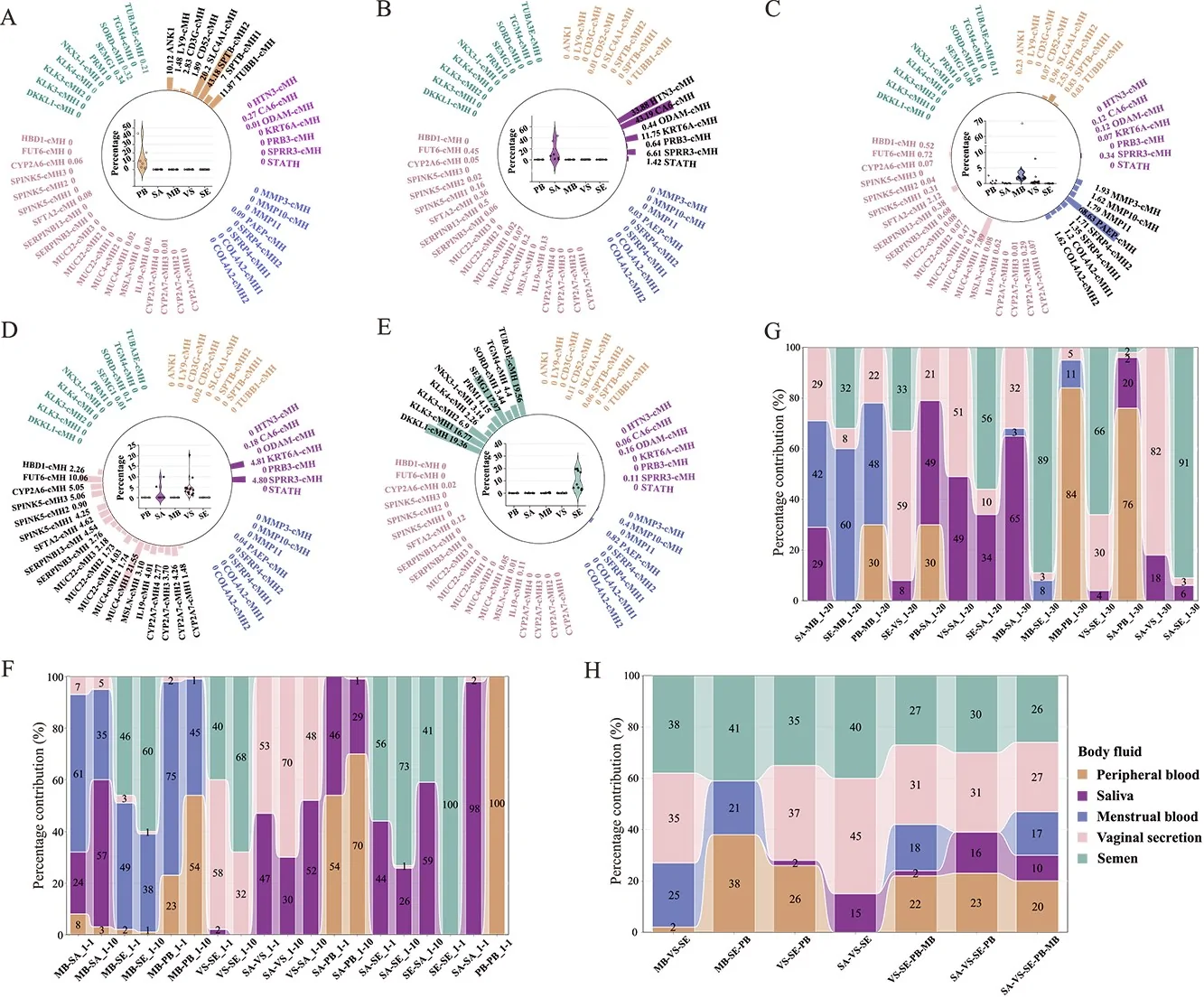
A–E: Percentage of average reads per marker across each single-source body fluid type (total reads per marker / total reads per body fluid type). Specifically: (A) peripheral blood; (B) saliva; (C) menstrual blood; (D) vaginal secretion; (E) semen. In the composite charts, the numerical labels on the outer ring represent the percentage of average reads (%), while the central violin plot displays the distribution of these values. F–H: Percentage contribution (%) of specific read counts for each body fluid component in the mixtures. Specifically: (F) typical-ratio binary mixtures (1:1 and 1:10); (G) highly imbalanced binary mixtures (1:20 and 1:30); (H) three- to five-component mixtures at equal ratios. The prefixes and suffixes in the mixture designations represent the body fluid components and their corresponding proportions, respectively. Abbreviations: PB, peripheral blood; SA, saliva; MB, menstrual blood; VS, vaginal secretion; SE, semen.

The panel contains 20 specific markers for VS, the largest number among five body fluid types. Results showed that these 20 markers contributed 90.04% of the total reads for VS samples, with ~9.79% arising from cross-expression with SA-specific markers (Fig. 2D). For SE stains, the 10 SE-specific markers accounted for 97.97% of the total reads, with only 2.03% of the reads attributed to weak false-positives (Fig. 2E). Specifically, low-level expression of the MB-specific markers MMP10-cMH and PAEP-cMH, the SA-specific marker ODAM-cMH, and the PB-specific marker CD52-cMH was detected in only a few SE samples, accounting for 0.40%, 0.82%, 0.16%, and 0.11% of the total reads in all SE samples, respectively. Similarly, weak cross-expression of *MMP10* and *CD52* genes in SE samples has been frequently reported in prior studies [3, 11, 47]. In summary, the body fluid-specific genes included in the panel developed in this study demonstrated robust specificity. These findings provide a solid experimental foundation for achieving high-accuracy BFID in complex forensic biological stains.

### Component characterization and donor attribution of mixture

Mixed stains are among the most common types of biological evidence found at crime scenes, typically deposited during struggles between suspects and victims or left behind by multiple perpetrators. For a long time, mixture deconvolution has remained a primary focus and a formidable challenge in forensic genetics research. Therefore, in this study, to comprehensively evaluate the deconvolution performance of the panel, mixtures of various types and different mixing ratios were prepared by mixing total RNA extracts from distinct body fluids.

#### Body fluid-specific gene expression profiling of binary to quinary mixtures

For mixtures, manual deconvolution primarily identifies the corresponding body fluid components by determining the percentage contribution of specific marker reads within the samples. To enhance the accuracy of mixture component characterization, we established type-specific cross-expression decision thresholds for each body fluid type. When the percentage of specific reads falls below the corresponding threshold, the corresponding body fluid component is considered absent, as these reads should be attributed to background noise arising from DNA contamination or cross-expression from non-target genes. Specifically, the decision thresholds for PB, SA, MB, VS, and SE were 0.5%, 1.5%, 0.8%, 0.9%, and 1.6%, respectively. Detailed information regarding these thresholds is presented in Supplementary Note S3 available online at http://bib.oxfordjournals.org/.

In 16 binary mixtures with distinct donors and body fluid types, prepared at typical ratios, the expected body fluid components were successfully detected. Figure 2F illustrates the proportional distribution of specific reads for each body fluid component within the mixtures. For mixtures with the same body fluid type combination, a 10-fold increase in a component's input ratio led to a corresponding but not strictly proportional rise in the percentage of its specific reads. Furthermore, when the total RNA inputs of two body fluid components were equal, their respective specific reads did not exhibit the expected 1:1 ratio, particularly for the SA component. We speculate that possible reasons involve the varying initial RNA template content of each component in the mixture and the differential amplification efficiency of their primers. Another factor may be the high proportion of microbial RNA within the total SA-derived RNA, leading to a significant deviation in the measured percentage contribution of SA-specific reads [7, 10]. Notably, when mixtures contained a MB component, low proportions of VS- and PB-specific reads were typically observed (Fig. 2F), as anticipated. In contrast, in the mixtures composed of any other two body fluid types, generally only the expected specific reads were detected. For two-component mixtures of the same body fluid type from different donors, the results reflected the characteristics of single-source samples, except for the SA–SA mixture, which exhibited a low proportion of VS-specific reads due to cross-expression (Fig. 2F). Figure 2G illustrates the expression profiles of 14 highly imbalanced mixtures. The results showed that minor components were successfully detected even at extreme mixture ratios of 1:20 and 1:30. In these mixtures, the body fluid serving as the major component consistently accounted for the vast majority of specific read counts. Conversely, although the minor component generated a lower percentage of read counts, it remained robustly detectable. In seven complex mixtures composed of three to five components in equal mixing ratios, all expected body fluid-specific reads were observed, with SE and VS showing higher percentage contributions (Fig. 2H). Furthermore, a very low proportion (2%) of SA-specific reads was observed in one ternary and one quaternary mixture containing VS but lacking SA. Further analysis traced these reads to cross-expression, primarily from the SA-specific markers KRT6A-cMH and SPRR3-cMH. These findings indicate that the panel can reliably characterize the components of highly imbalanced binary and complex mixtures.

#### Donor attribution in mixtures using cMH analysis

Following the deconvolution of body fluid components within mixtures, the correct attribution of each component to its respective donor using cMH genotypic information is required. Because the specific markers of each component in a mixture originate solely from the corresponding donor, the resulting cMH profile matches that of the corresponding donor. For instance, in a SE–SA mixture, the SE-specific cMHs solely reflect the genotype of the SE donor and do not provide information regarding the SA donor. Furthermore, considering the co-expression of PB- and VS-specific genes in MB samples, the corresponding cMHs can similarly be employed for MB donor attribution. In this study, only the 48 cMHs distributed across 36 body fluid-specific genes were utilized for donor attribution. The remaining five genes, lacking cMHs, were employed exclusively for BFID.

Detailed cMH genotyping results for binary mixtures, comprising distinct body fluid types from different donors at typical mixture ratios (1:1 and 1:10), are presented in Supplementary Table S3. In the MB–SA mixtures (Supplementary Table S3-1), five MB cMHs and three SA cMHs were successfully used to exclude non-contributors. Genotype dropout of COL4A2-cMH1 was observed when MB was the minor component (MB:SA = 1:10). Additionally, four PB- and VS-specific cMHs were detected, which could assist in excluding the SA contributor. For the MB–SE mixtures (Supplementary Table S3-2), four MB cMHs and six SE cMHs enabled the exclusion of non-contributors and the correct attribution of the contributors. Similarly, five PB- and VS-specific cMHs from the MB component were detected in the 1:1 MB–SE mixtures, which could exclude the SE contributor. Given the typical co-expression of PB-specific genes in MB, read counts generated by PB-specific cMHs from the MB component in MB–PB mixtures may shift the allelic balance of PB-specific cMHs from the PB component, potentially producing a mixed genotype profile. In this study, three MB cMHs and three PB cMHs in MB-PB mixtures at typical ratios exhibited genotypes inconsistent with those of non-contributors (Supplementary Table S3-3). Although all PB cMH genotypes within the mixtures were consistent with the PB contributor, noticeable shifts in the allelic balance were observed for CD52-cMH and SLC4A1-cMH (Fig. 3A). Specifically, compared to the single-source PB sample from the same contributor, the allelic balance shifts for CD52-cMH and SLC4A1-cMH in the mixture were ≥ 0.05. This shift became increasingly pronounced as the proportion of the PB component decreased, a trend further confirmed in highly imbalanced mixtures. For example, at a MB:PB ratio of 20:1, the allelic balance of the PB cMHs shifted substantially, even manifesting as mixed genotypes (Fig. 3B). Such occurrences may mislead forensic practitioners into assuming the presence of an additional PB contributor in the mixture. Consequently, in forensic practice, extreme caution is warranted during the deconvolution of highly imbalanced mixtures dominated by MB. In the remaining typical ratio binary mixtures, the two components collectively yielded a minimum of six specific cMHs that were genotypically discordant with the non-contributors, thereby facilitating their exclusion (Supplementary Table S3-4 to S3-7). In highly imbalanced mixtures (1:20 and 1:30 ratios), despite one or two marker drop-outs in the minor component, the remaining markers enabled the reliable exclusion of non-contributors. The marker detection profiles for all highly imbalanced mixtures are detailed in Supplementary Table S4. Given that our preceding analysis revealed low-level expression of certain SA-specific genes in VS and vice versa, we further investigated the impact of non-specific reads generated by cross-expression on the allelic balance of cMHs within SA–VS mixtures. As illustrated in Fig. 3C–F, when the mixing ratios shifted gradually from 1:1 to 1:30, the non-specific reads generated by the major component affected the allelic balance of the minor component-specific cMHs to varying degrees, and even resulting in mixed genotype profiles. Consequently, forensic practitioners should carefully evaluate the underlying causes when encountering mixed genotypes for body fluid-specific markers in casework.

**Figure 3:**
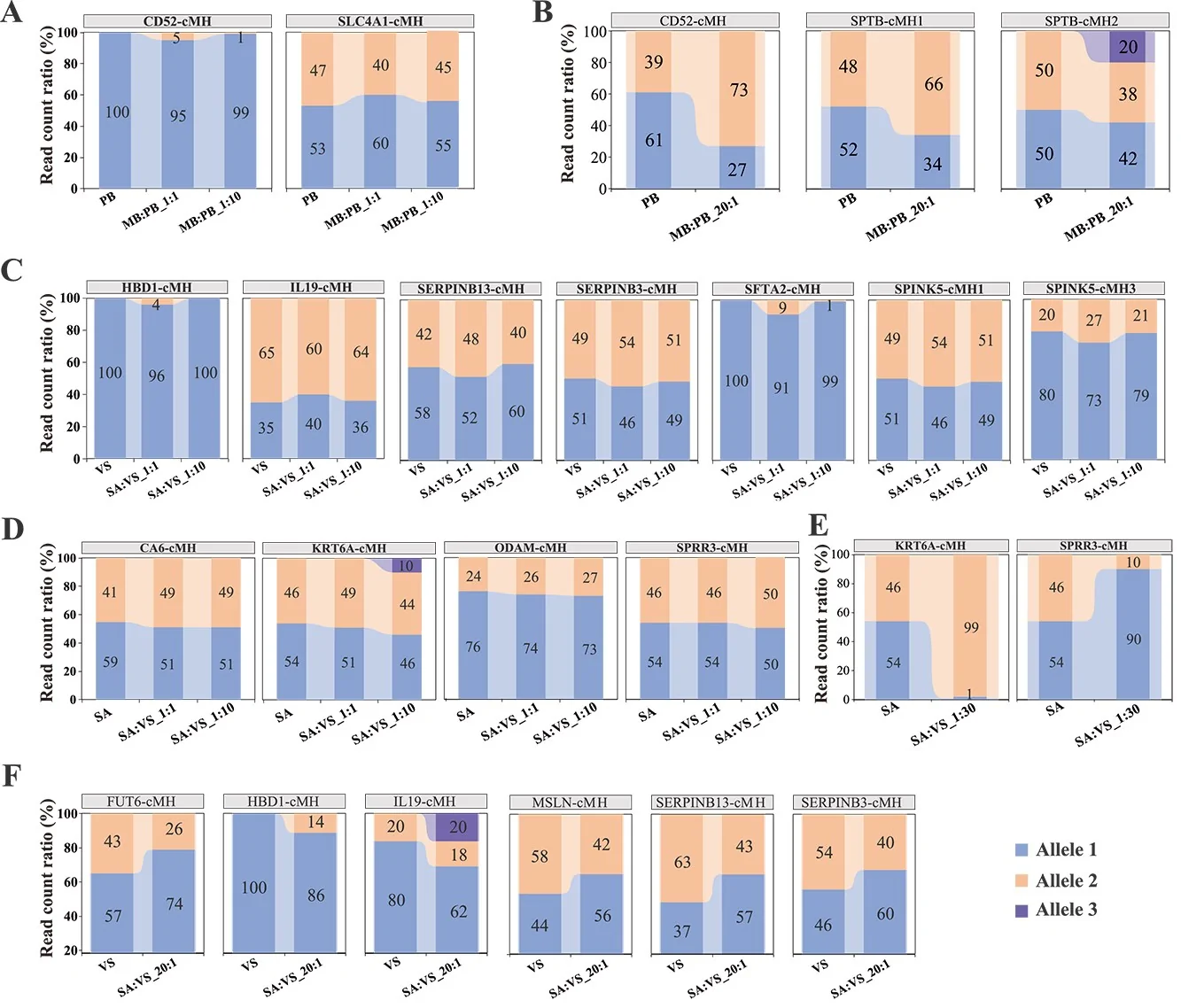
Significant allelic balance shifts of cMHs observed in binary mixtures. A–B: Allelic balance shifts of blood-specific cMHs in MB–PB mixtures. C–F: Allelic balance shifts of cMHs in SA–VS mixtures. Alleles are color-coded (Allele 1, light blue; Allele 2, light orange; Allele 3, dark purple). Abbreviations: PB, peripheral blood; SA, saliva; MB, menstrual blood; VS, vaginal secretion.

For binary mixtures composed of the same body fluid type from two different contributors, the cMH profiles displayed clear mixed genotyping characteristics (Supplementary Fig. S3A–B). In conventional DNA profiling, for a 1:1 mixture involving a heterozygous donor and a homozygous donor sharing one allele, the expected allelic count ratio (ACR) is typically 3:1. However, we found that some cMHs in RNA profiling exhibited severe allelic imbalance in 1:1 mixtures, particularly DKKL1-cMH (ACR = 9:1) in the SE–SE mixture (Supplementary Fig. S3B). We hypothesize that this phenomenon may be attributed to significant inter-individual variation in gene expression levels. Consequently, unlike gDNA profiling, the ACR in RNA profiling is difficult to use as a reliable basis for inferring mixing proportions of components in mixtures [10]. Furthermore, we evaluated the deconvolution efficacy of the panel in complex mixtures comprising three to five components. And the results indicated that cMH typing profiles in these mixtures exhibited greater complexity. Specifically, as the complexity of mixtures increased, a few cMHs experienced marker or allele drop-out (Supplementary Fig. S4). We hypothesize this was primarily driven by competitive primer inhibition or stochastic effects during PCR amplification. Additionally, minor allelic imbalance and allele drop-in events were observed in PB-, SA-, and VS-specific cMHs. As previously discussed, this may stem from the cross-expression of body fluid-specific genes in certain non-target body fluid components (e.g., MB and SA). For instance, in the MB–VS–SE mixture, the non-specific reads from the MB component substantially shifted the allelic balance of VS cMHs, ultimately resulting in allele drop-out, drop-in, and allelic imbalance (Supplementary Fig. S4). In summary, donor attribution in complex mixtures is a highly multifactorial process, remaining a formidable challenge in forensic identification. Detailed cMH genotyping results for all complex mixtures are provided in Supplementary Table S5.

### Construction of machine learning-based classification models for BFID

To evaluate the applicability and efficacy of the panel, we constructed classification models based on various machine learning algorithms to enable accurate BFID. Importantly, the machine learning models were solely employed to predict the presence of body fluid components, whereas the attribution of these components to contributors relied on the genotypic information of the cMH markers. For model development, all amplicon reads from the panel were included. During the model evaluation phase, we systematically assessed the performance of each classification model using a combination of metrics. Furthermore, SHAP analysis was employed to identify the key feature variables driving the models' classification decisions.

#### Identification of single-source body fluid stain using multi-class machine learning models

Transcriptome sequencing data are typically characterized by high dimensionality, non-linearity, and individual heterogeneity. Based on an ensemble learning mechanism, the RF algorithm effectively processes high-dimensional sparse data and exhibits strong robustness against noise. Furthermore, by maximizing the classification margin, the SVM algorithm demonstrates robust generalization capability and predictive stability in small-sample, high-dimensional classification tasks. Therefore, we initially constructed RF and SVM multiclass models to preliminarily validate the BFID efficacy of the dual-purpose panel. Prior to model construction, PCA was performed on all single-source body fluid samples to assess potential batch effects. As shown in Supplementary Fig. S5, samples clustered distinctly according to body fluid types rather than by sequencing batch, indicating the absence of significant batch effects and confirming the reliability of the dataset.

Figure 4 illustrates the prediction results of the RF multi-class model on the independent test set. The results demonstrated that the model exhibited strong classification performance, accurately classifying all unknown single-source body fluid samples (Fig. 4A). Specifically, both the AUROC and the area under the PRC (AUPRC) reached 1.00 for each body fluid type (Fig. 4B and C), with the Macro F1-score of 1.00. Detailed evaluation metrics regarding the predictive performance for each body fluid type are provided in Supplementary Table S6-1. In addition, the accuracy-based learning curve indicates that the current sample size is sufficient for the model's predictive performance to reach convergence (Fig. 4D). These findings demonstrate that the model's strong performance is robust and not an artifact of overfitting. Furthermore, the SHAP global feature importance bar plot (Fig. 4E) displays the top 20 global features with the highest contributions to the model's decisions, where CYP2A7-cMH4, STATH, and KLK3-cMH1 markers rank as the top three.

**Figure 4:**
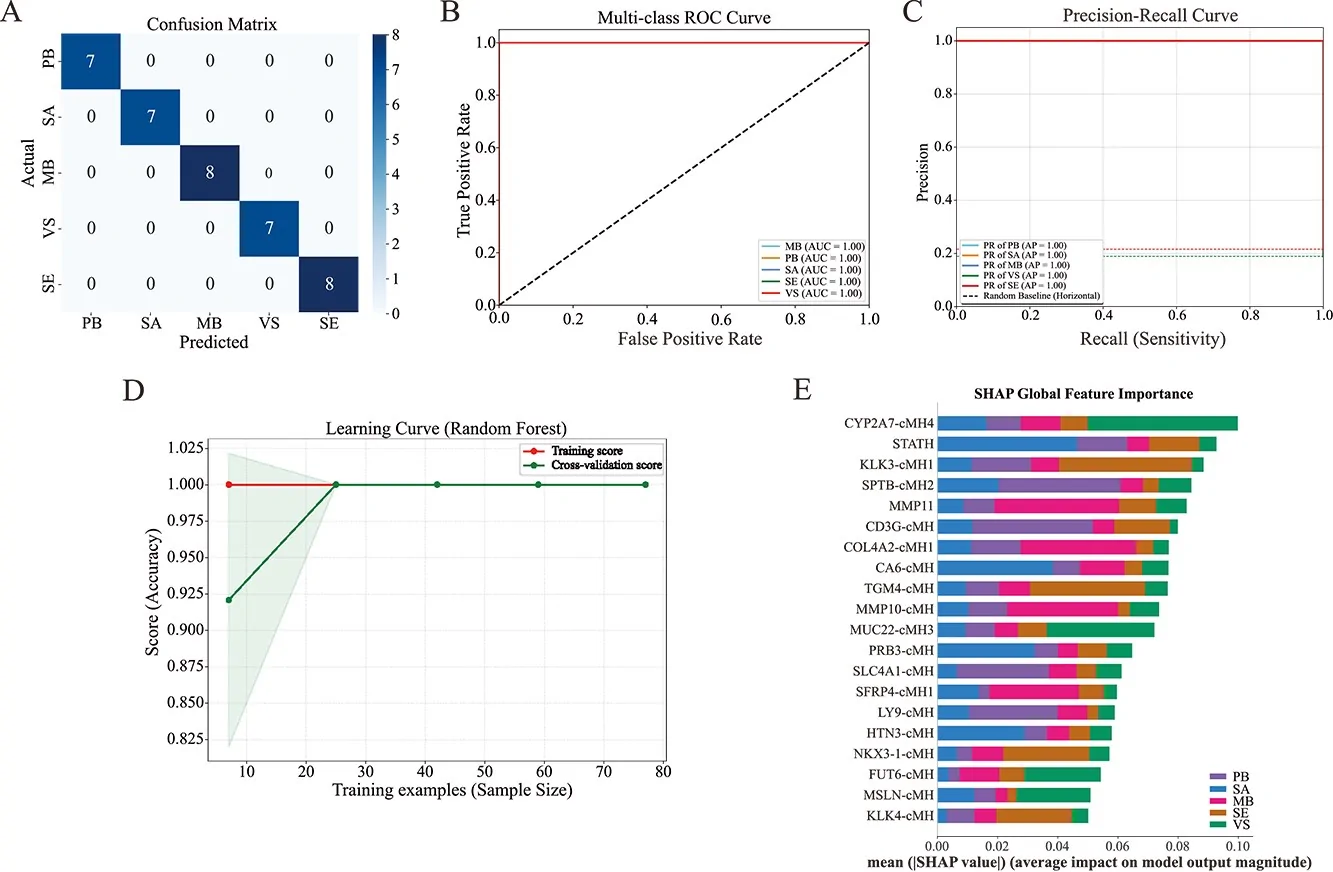
Performance evaluation and SHAP-based feature interpretation of the Random Forest (RF) multi-class model for single-source body fluid inference. A. Confusion matrix of the RF model on the test set. B. Multi-class ROC curves of the RF model, and AUC values for all body fluid types are 1.00. C. Precision-recall (PR) curves of the RF model, and average precision for each body fluid type is 1.00. D. Learning curve analysis of the RF model. The red line represents the training accuracy, while the blue line represents the cross-validation accuracy. The blue shaded area denotes the standard deviation of the cross-validation accuracy. E. SHAP global feature importance bar chart showing the top 20 contributing features. Bar lengths represent the mean absolute SHAP values (|SHAP value|). Abbreviations: PB, peripheral blood; SA, saliva; MB, menstrual blood; VS, vaginal secretion; SE, semen.

The SVM multi-class model demonstrated strong performance highly consistent with the RF model, accurately predicting all unknown single-source body fluid samples in the independent test set (Fig. 5A). The AUROC, AUPRC, and Macro F1-score of this model all reached 1.00, and the accuracy-based learning curve indicated that the SVM multi-class classifier had achieved full convergence (Fig. 5B–D). Supplementary Table S6-2 presents the detailed evaluation metrics regarding the predictive performance of the SVM model. To further explore the model's discriminative pattern within the feature space, we performed PCA and evaluated the SVM linear weights for the top 20 selected core features. The PCA results revealed a significant clustering effect for the five types of body fluid within a 2D projection space (Fig. 5E). Figure 5F displays the top 20 variables ranked by their linear weight coefficients, with HTN3-cMH, SPTB-cMH2, and STATH markers being the top three contributors. In summary, both the RF and SVM multi-class models developed in this study exhibited strong performance in identifying single-source body fluid stains, fully demonstrating the robust BFID capability of the panel. More importantly, through multifaceted approaches including SHAP attribution analysis, SVM linear weight evaluation, and PCA clustering visualization, we have thoroughly elucidated the predictive processes and decision-making logic of the models, thereby significantly enhancing their interpretability and transparency. The specific hyperparameter search spaces and optimal configurations for the two multi-class models are detailed in Supplementary Note S4 available online at http://bib.oxfordjournals.org/.

**Figure 5:**
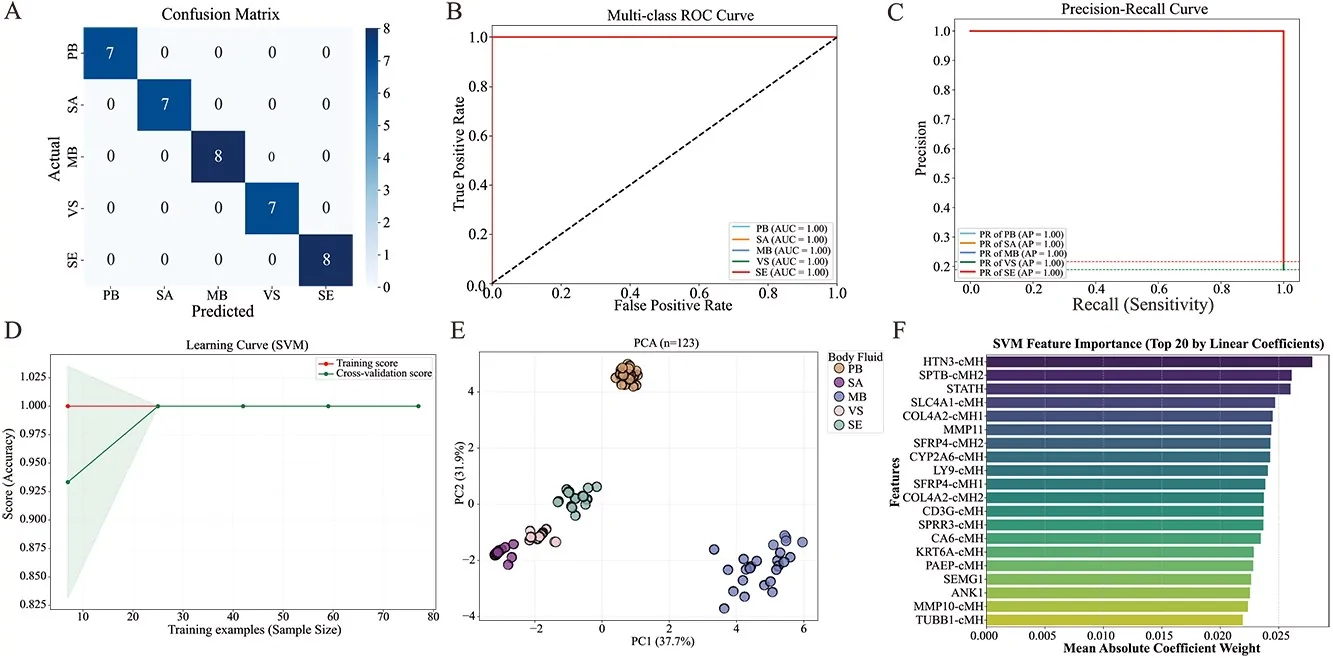
Performance evaluation and linear weights analysis of the Support Vector Machine (SVM) multi-class model for single-source body fluid inference. A. Confusion matrix of the model on the independent test set. B. Multi-class ROC curves and AUC values of the model. C. Precision-recall curves of the model. D. Learning curve analysis of the SVM model. The red line represents the training accuracy, while the green line represents the cross-validation accuracy. The green shaded area denotes the standard deviation of the cross-validation accuracy. E. Principal component analysis (PCA) of body fluid samples based on the top 20 informative features identified by the SVM model. F. Quantitative analysis of feature weights based on the linear coefficients of the SVM model. Abbreviations: PB, peripheral blood; SA, saliva; MB, menstrual blood; VS, vaginal secretion; SE, semen.

#### Development of multi-label classification models combined with an *in silico* mixing strategy for mixture component inference

Multi-class models constructed based on single-source body fluid sequencing data primarily establish decision boundaries for mutually exclusive classes. Consequently, they have difficulty capturing the overlapping features or weak signals in mixtures, making them prone to missed detections and misclassifications in multi-label identification tasks. Furthermore, preparing large-scale empirical mixtures that cover diverse body fluid combinations and mixing ratios is prohibitively expensive and operationally complex. In recent years, numerous studies have demonstrated that the *in silico* strategy serves as a highly efficient data augmentation approach in the life sciences [48, 49]. The simulated data generated through data enrichment strategies such as transfer learning, data augmentation, and bootstrapping effectively help overcome challenges like data scarcity and heterogeneity in empirical studies [50]. Therefore, we adopted an established *in silico* mixing strategy from computational biology to algorithmically synthesize simulated mixture expression profiles from real single-source data, aiming to explore its feasibility for BFID while compensating for the limited size and diversity of actual mixture datasets. Although the expression levels of body fluid-specific genes exhibit a non-linear relationship with sample volume, the simulated mixture data, generated by combining single-source body fluid data according to specific volume ratios and incorporating Gaussian noise and random feature dropout, can still effectively reflect the body fluid-specific expression characteristics of these genes. This ensures that the model learns expression patterns highly consistent with real mixtures, as the core mechanism of the machine learning classifier relies on capturing the specific, qualitative expression profiles of body fluid-specific genes. Additionally, we constructed various complex non-linear and simple linear models, and compared their predictive performance to ultimately identify the optimal classifier, aiming to provide an efficient and reliable tool for BFID.

Initially, to evaluate the reliability of the simulated mixture data and their ability to accurately reflect the characteristics of gene expression in real mixtures, we employed the t-SNE algorithm for dimensionality reduction and visualization. As shown in Fig. 6A, we first examined the global distribution relationship between the two data sources using a binary labeling strategy. The results revealed that the simulated and real data exhibited a high degree of overlap and intermingling in the feature space. This suggested that the simulated data do not deviate significantly from the real data. Furthermore, to verify the fidelity of the simulated data in representing the expression profiles of specific mixture types, we stratified the samples by mixture type (Fig. 6B). The visualization demonstrated that real and simulated samples belonging to the same mixture type consistently clustered tightly together. This high intra-class consistency confirmed that the simulation strategy not only fits the real data at a macroscopic level but also preserves the specific gene expression characteristics inherent to different mixture types, supporting the utility of the simulated data for downstream model construction.

**Figure 6:**
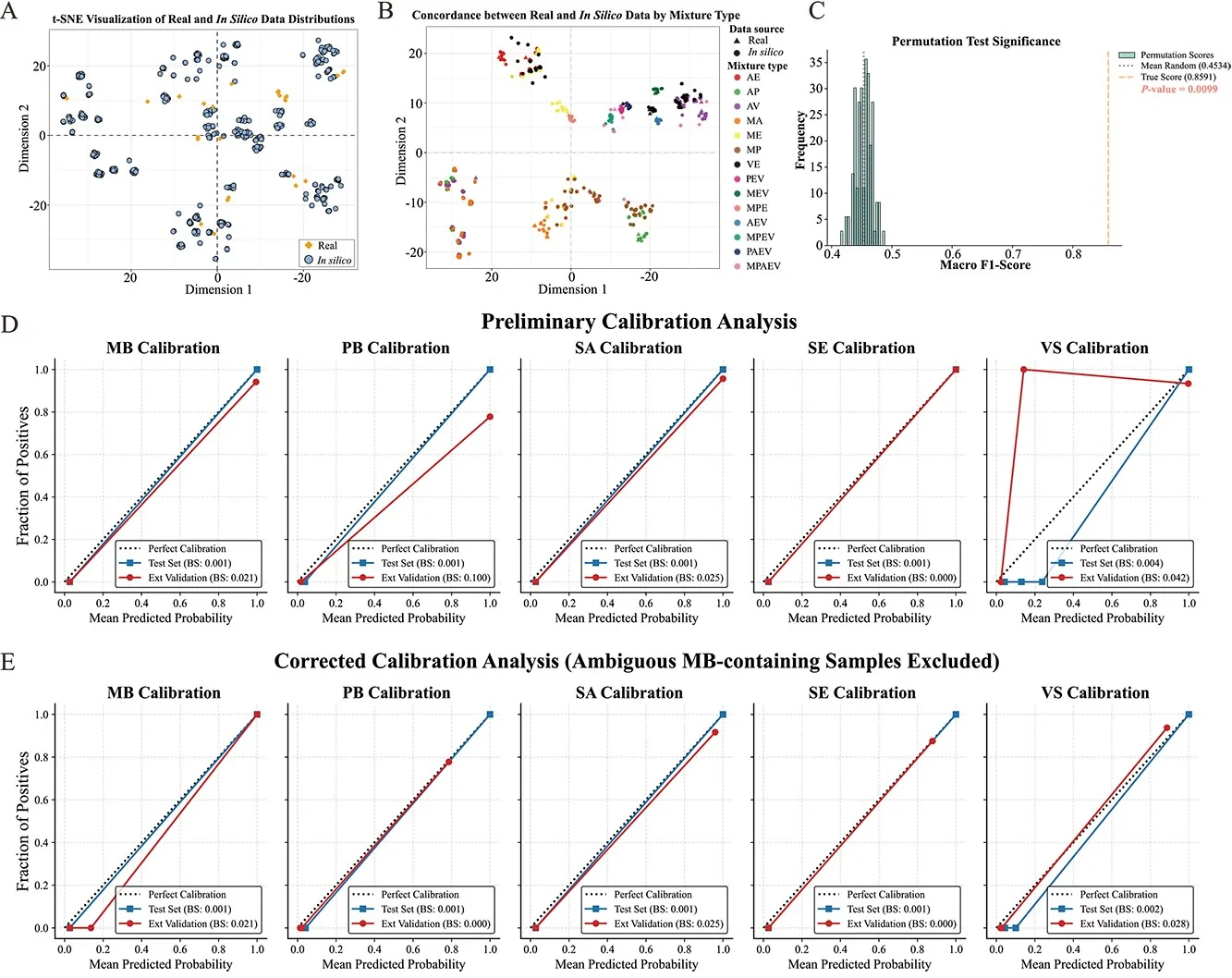
A. T-SNE visualization of global sample distribution based on data source (real versus *in silico*). B. T-SNE visualization of both data sources stratified by mixture type. C. Permutation test results demonstrating the statistical significance of the SVM-RFE model. D. Probability calibration curves of the SVM-RFE model based on all samples from the independent test and external validation sets. E. Corrected probability calibration curves of the SVM-RFE model for the remaining samples in the independent test and external validation sets, following the exclusion of ambiguous MB-containing samples (misclassified due to predict PB or VS components). Abbreviations: P/PB, peripheral blood; A/SA, saliva; M/MB, menstrual blood; V/VS, vaginal secretion; E/SE, semen.

Using the 946 expression profiles in the final training set, we employed 10-fold cross-validation coupled with a grid search strategy to determine the optimal hyperparameters for each model and construct the corresponding multi-label classification model. In addition, for each model, we established the optimal class-specific decision threshold for each body fluid type. Initially, we evaluated the models based on their prior-informed classification performance on both the independent test and external validation sets. On the independent test set (Table 2), the SVM-RFE and XGBoost models exhibited the optimal performance, with both achieving an EMR, Macro AUC, and Macro F1-score of 1.00. This demonstrated that for unknown single-source body fluid stains, multi-label classification models constructed synergistically with *in silico* mixing strategies achieved equivalent efficacy to the multi-class models based on authentic empirical data. These were followed sequentially by the RF, Elastic Net LR, and KNN models. On the external validation set, the SVM-RFE and Elastic Net LR models exhibited the best performance (Table 3). The SVM-RFE model achieved an EMR of 0.95 (95% CI: 0.88–1.00), a Macro AUC of 0.98 (95% CI: 0.96–1.00), and a Macro F1-score of 0.99 (95% CI: 0.97–1.00). Closely matching this, the Elastic Net LR model yielded an EMR of 0.95 (95% CI: 0.88–1.00) and a Macro AUC of 0.99 (95% CI: 0.98–1.00), with a Macro F1-score of 0.99 (95% CI: 0.97–1.00). This was followed by the XGBoost model, which also achieved a high EMR of 0.93 (95% CI: 0.83–1.00). Notably, the performance of the RF model on the external validation set showed a significant decline compared to the independent test set, yielding an EMR of only 0.83 (95% CI: 0.78–0.88). Additionally, as shown in Supplementary Note S5 available online at http://bib.oxfordjournals.org/, the raw classification performance of all models exhibited a slight decrease compared to their prior-informed classification performance. Specifically, on the independent test set, the raw EMRs for the SVM-RFE, XGBoost, and Elastic Net LR models were 0.91, 1.00, and 0.82, respectively. On the external validation set, the raw EMRs for these three models were 0.83, 0.85, and 0.85, respectively. By comparison, the raw classification performance of the RF and KNN models was slightly inferior to that of the three aforementioned models. The detailed evaluation metrics of the raw classification performance of all models, together with comparisons between the raw and prior-informed classification performance, are provided in Supplementary Note S5 available online at http://bib.oxfordjournals.org/. Based on the comprehensive findings, the three top-performing models (SVM-RFE, XGBoost, and Elastic Net LR) were selected for pairwise McNemar's tests on prior-informed predictions to assess whether the performance differences among the models were statistically significant. The results indicated that no significant performance differences existed among the models on both datasets (all *P* > 0.017, Bonferroni-corrected threshold: 0.05/3 = 0.017; see Supplementary Note S6 available online at http://bib.oxfordjournals.org/ for details). Although the XGBoost model exhibited slightly superior raw classification performance, in forensic practice, the prior-informed classification performance more accurately reflects a model's practical value when handling specific body fluid types (e.g., MB). The masking procedure effectively mitigates the "false positive" interference caused by biological co-expression signatures, thereby significantly reducing the misclassification of MB components. Therefore, taking into account the overall performance and generalization stability of all models on both datasets, the SVM-RFE multi-label classifier was ultimately identified as the optimal model for subsequent analysis.

**Table 2:** Prior-informed classification performance metrics of five multi-label classification models on the independent test set.

Model	Exact match ratio	Macro AUC	Hamming loss	Macro precision	Macro recall	Macro F1-score
KNN	0.84 (0.74–0.95)	1.00 (−)	0.03 (0.01–0.05)	0.89 (0.82–0.96)	1.00 (−)	0.94 (0.87–0.98)
Elastic Net LR	0.92 (0.84–1.00)	1.00 (−)	0.03 (0.00–0.06)	0.88 (0.76–1.00)	1.00 (−)	0.94 (0.85–1.00)
RF	0.95 (0.87–1.00)	1.00 (−)	0.01 (0.00–0.03)	1.00 (−)	0.95 (0.86–1.00)	0.97 (0.91–1.00)
SVM-RFE	1.00 (−)	1.00 (−)	0.00 (0.00–0.00)	1.00 (−)	1.00 (−)	1.00 (−)
XGBoost	1.00 (−)	1.00 (−)	0.00 (0.00–0.00)	1.00 (−)	1.00 (−)	1.00 (−)

Note: For metrics achieving a perfect score of 1.00 (e.g. Exact Match Ratio, Macro AUC, Macro F1-score), the 95% confidence intervals are not reported, as traditional variance-based or bootstrap estimations converge to a point estimate of zero variance due to the ceiling effect in the independent test set (*n* = 37). Abbreviations: KNN, *K*-Nearest Neighbors; Elastic Net LR, Elastic Net Logistic Regression; RF, Random Forest; SVM-RFE, Support Vector Machine-Recursive Feature Elimination; XGBoost: eXtreme Gradient Boosting.

**Table 3:** Prior-informed classification performance metrics of five multi-label classification models on the external validation set.

Model	Exact match ratio	Macro AUC	Hamming loss	Macro precision	Macro recall	Macro F1-score
KNN	0.73 (0.58–0.85)	0.98 (0.95–1.00)	0.07 (0.04–0.11)	0.99 (0.96–1.00)	0.85 (0.78–0.92)	0.92 (0.87–0.96)
Elastic Net LR	0.95 (0.88–1.00)	0.99 (0.98–1.00)	0.01 (0.00–0.03)	0.98 (0.95–1.00)	1.00 (−)	0.99 (0.97–1.00)
RF	0.83 (0.78–0.88)	0.99 (0.97–1.00)	0.06 (0.05–0.08)	1.00 (−)	0.83 (0.76–0.89)	0.91 (0.87–0.94)
SVM-RFE	0.95 (0.88–1.00)	0.98 (0.96–1.00)	0.01 (0.00–0.03)	0.98 (0.94–1.00)	1.00 (−)	0.99 (0.97–1.00)
XGBoost	0.93 (0.83–1.00)	0.99 (0.98–1.00)	0.02 (0.00–0.04)	1.00 (−)	0.97 (0.93–1.00)	0.98 (0.96–1.00)

Note: As detailed in Table 2, the 95% confidence intervals for metrics achieving a perfect score of 1.00 are omitted. Abbreviations: KNN, *K*-Nearest Neighbors; Elastic Net LR, Elastic Net Logistic Regression; RF, Random Forest; SVM-RFE, Support Vector Machine-Recursive Feature Elimination; XGBoost: eXtreme Gradient Boosting.

In summary, even when trained on limited real data combined with large-scale simulated data, the multi-label classification models exhibited strong efficacy for BFID. This indicates that the *in silico* mixing strategy can serve as an effective approach to overcome the limitations of insufficient numbers of mixtures and limited component diversity in empirical studies.

#### Systematic performance evaluation and robustness validation of the optimal multi-label classifier

The hyperparameter search space and optimal configuration of the SVM-RFE multi-label classifier are detailed in Supplementary Note S7 available online at http://bib.oxfordjournals.org/. The optimal class-specific decision thresholds for the five body fluid types were determined as follows: PB (0.438), SA (0.463), MB (0.677), VS (0.135), and SE (0.710). Following RFE screening, the optimal number of retained features for each class was uniformly fixed at 15. Additionally, the confusion matrix and detailed predictive outcomes of the SVM-RFE multi-label classifier on the independent test set are displayed in Supplementary Table S7-1 and Supplementary Fig. S6, respectively, with the latter illustrating the probability-based decision inference process guided by the optimal decision thresholds. Meanwhile, the exhaustive results for the external validation set are presented in Supplementary Table S7-2. To rule out overfitting and verify the genuine association between the selected features and type labels, a permutation test was performed based on the training dataset (Fig. 6C). The results revealed that the classification score achieved by the SVM-RFE multi-label classifier on the real dataset (Macro F1-score = 0.8591) was significantly higher than that in the null distribution generated by random label permutations (Macro F1-score = 0.4543, *P* = 0.0099). This statistically confirms that the classifier successfully captured real specific signatures, rather than relying on random noise or chance. In addition, to further evaluate the reliability of the classifier's output probabilities, probability calibration curves were plotted, and Brier scores (BS) were calculated for both the independent test and external validation sets. In the preliminary calibration analysis (Fig. 6D), the calibration curves for PB and VS in the external validation set exhibited noticeable deviations, accompanied by relatively high BS values. This result was hypothesized to result from the inability to apply masking processes (detailed in Section 2.6) to the output labels of MB-containing samples during probability calibration analysis, which consequently led to predictive outputs containing PB or VS components being erroneously categorized as misclassifications. To eliminate interference caused by the co-expression of VS- and PB-specific genes in MB, the nine MB-containing samples that were misclassified due to the predicted presence of PB or VS components were excluded from the PB and VS calibration assessment. The corrected results demonstrated that the classifier exhibited strong probability calibration across both datasets (Fig. 6E). Specifically, the calibration curves for all body fluid types aligned tightly with the ideal diagonal line, accompanied by a marked decrease in BS values. These findings substantiate that the predictive probabilities generated by the SVM-RFE multi-label classifier possess high statistical reliability, and that the classifier is free from probability overfitting.

To further validate its generalization ability, the SVM-RFE multi-label classifier was evaluated on sensitivity test samples (*n* = 25) and degraded samples (*n* = 26), achieving high predictive accuracy on both sample sets (see the corresponding sections below). Finally, to rigorously assess the stability of the classifier, a feature ablation study was conducted using SHAP analysis. The class-specific SHAP feature importance rankings are provided in Supplementary Fig. S7. After eliminating the core feature with the highest attribution weight for each respective class, the classifier was reconstructed using the remaining features. Re-evaluation of the independent test and external validation sets using the reconstructed classifier (detailed in Supplementary Note S8 available online at http://bib.oxfordjournals.org/) yielded EMR values of 0.95 (95% CI: 0.87–1.00) and 0.95 (95% CI: 0.88–1.00), respectively, and Macro F1-scores of 0.98 (95% CI: 0.93–1.00) and 0.99 (95% CI: 0.97–1.00), respectively. Collectively, these results demonstrated that the SVM-RFE classifier exhibits robust generalization, maintaining high predictive accuracy even in challenging scenarios involving the loss of primary markers.

### Reproducibility and sensitivity analyses of the dual-purpose assay

We evaluated the assay's technical stability by assessing the read count distributions and detection consistency of markers across triplicate analyses of single-source body fluid samples. As illustrated in Fig. 7A, the read count distributions of the body fluid-specific markers exhibited minimal variations across the three technical replicates. Furthermore, marker detection remained highly consistent across the replicate runs (Supplementary Table S7-3). Collectively, these findings substantiate the high reproducibility and technical stability of the assay.

**Figure 7:**
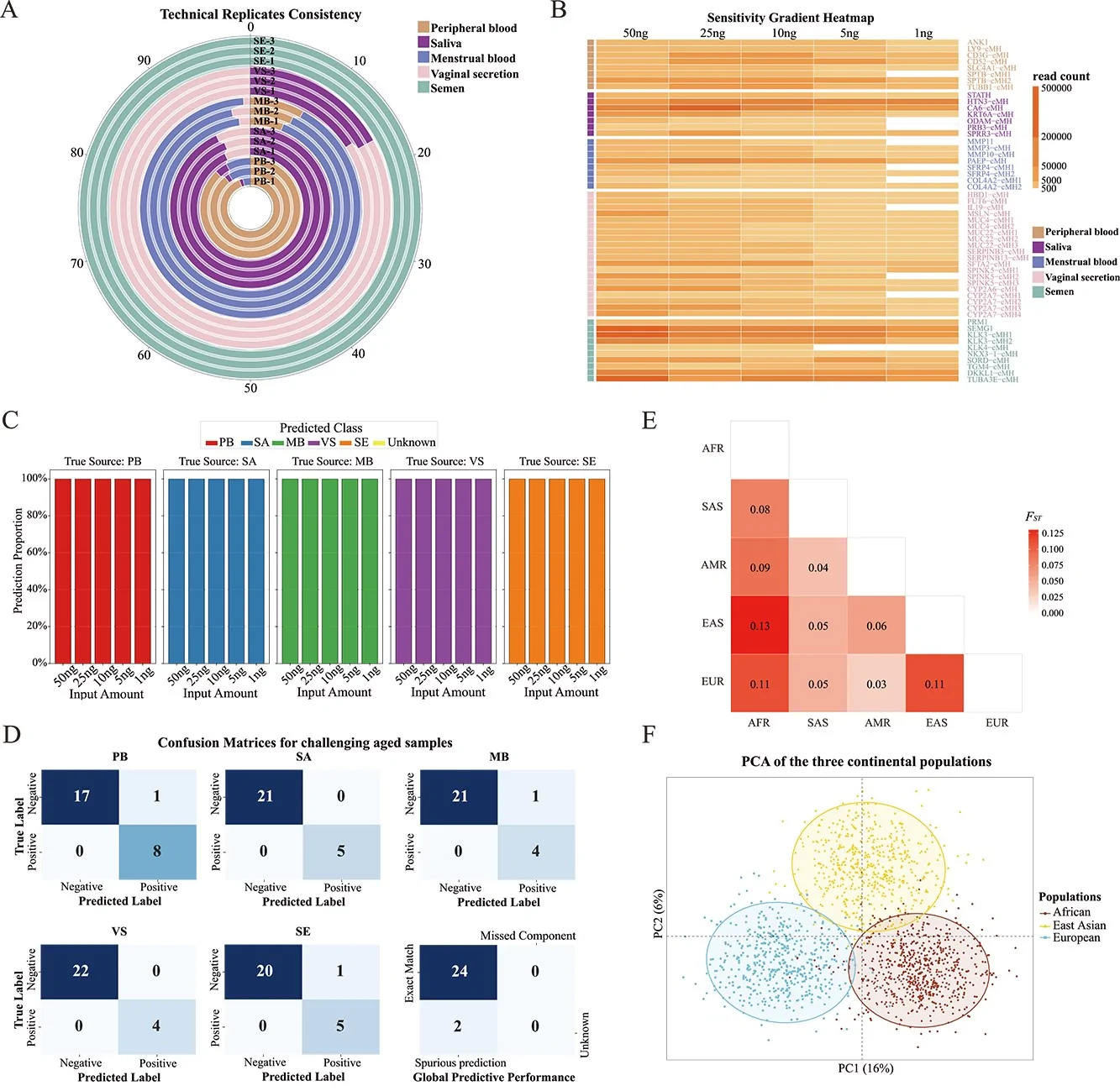
A. Results of triplicate runs for each body fluid sample. Circular bar lengths indicate the proportion of body fluid-specific reads out of the total sample reads. B. Heatmap of the sensitivity gradient. The color of the cells, ranging from white to dark orange, represents a gradual increase in amplicon read counts. C. Stacked bar plots illustrating the prediction composition of the SVM-RFE multi-label classifier across varying RNA input levels, categorized by true body fluid source. Colors correspond to the predicted class labels. D. Confusion matrices of the SVM-RFE multi-label classifier for challenging aged samples. E. *F_ST_* values among five continental populations. The color gradient of the heatmap, ranging from white to red, represents a gradual increase in *F_ST_* values. F. Principal component analysis (PCA) results based on the genotype data of 48 cMHs for individuals from African (AFR), East Asian (EAS), and European (EUR) populations. Abbreviations: PB, peripheral blood; SA, saliva; MB, menstrual blood; VS, vaginal secretion; SE, semen.

According to previous studies, a total RNA input of 20–25 ng has been suggested to be optimal for maintaining a balance between the detection sensitivity of body fluid-specific markers and mitigating cross-expression. Consequently, a total RNA input of 25 ng was employed for all other routine assays in this study. To determine the limit of detection (LOD) of the panel, we systematically evaluated the sensitivity of each marker across a range of total RNA inputs (50 ng, 25 ng, 10 ng, 5 ng, and 1 ng). A heatmap of sensitivity across the total RNA input range, illustrating the read counts and detection status of all markers, is shown in Fig. 7B. The results indicated that as the total RNA input gradually decreased, a few markers did not exhibit the expected reduction in read counts. We attribute this phenomenon to variations in reverse transcription efficiency or subtle fluctuations during library normalization and quantification. Nevertheless, amplicon drop-out was consistently observed at lower total RNA input levels. For PB samples, only SPTB-cMH1 was undetected when the total RNA input was as low as 1 ng (Fig. 7B). In contrast, at 1 ng total RNA input, two markers (ODAM-cMH and PRB3-cMH) in SA samples failed to be detected. Given that these two targets consistently exhibit relatively low read counts in routine assays, we speculate that this result is attributable to suboptimal primer amplification efficiency or inherently low gene expression levels. By contrast, the read counts of HTN3-cMH and CA6-cMH still exceeded 5 × 10^5^. For the eight MB markers, three were undetected at 1 ng total RNA input. VS, as the body fluid type with the largest number of specific markers, also had only two markers undetected at the minimal RNA template input, maintaining a high marker detection rate. SE-specific markers demonstrated the highest sensitivity among all body fluid types, as nine of the ten markers were successfully detected at 1 ng total RNA input, and seven of them still maintained high read counts (>16 000). Overall, the panel developed in this study exhibited adequate sensitivity at the LOD. Most specific markers were detectable at a low total RNA input of 1 ng, which facilitates the detection of trace samples in practice.

Regarding the six novel body fluid-specific genes identified in this study, the results demonstrated that these genes were robustly detected across all three technical replicates. At a total RNA input of 1 ng, marker drop-out was observed only for COL4A2-cMH1, while the remaining markers were successfully detected. These findings indicate that these six novel genes exhibit high detectability at low RNA input. Furthermore, when all sensitivity test samples were evaluated using the SVM-RFE multi-label classifier, the model correctly classified all samples at all five total RNA input levels in both the raw and prior-informed prediction outcomes (Fig. 7C). This indicates that the model maintains robust generalization even with partial marker drop-out at a total RNA input of 1 ng.

### Efficacy evaluation of the dual-purpose panel on challenging aged samples

In forensic casework, biological stains recovered from crime scenes are frequently deposited on diverse substrates and subjected to varying degrees of aging and degradation. Therefore, it is essential to evaluate the detection performance of the panel when challenged with extensively aged samples deposited on different substrates. The detection profiles of all indoor-aged samples are presented in Supplementary Fig. S8. For the PB swab with the longest storage time (346 days), all PB-specific markers were robustly detected except for SPTB-cMH1. In SA stains deposited on paper tissues and aged for 3 and 200 days, ODAM-cMH was detected in the 3-day sample but not in the 200-day sample. Similarly, in a MB swab aged for 203 days and a VS swab aged for 97 days, only one and two specific markers were not detected, respectively. Furthermore, 200 μL aliquots of semen deposited on condoms and cotton swabs were aged for 104 days and two years, respectively, with one to two specific markers undetected in each sample. To further investigate the panel's efficacy in particularly challenging scenarios, we additionally prepared separate groups of degraded samples, each exposed to one of the following conditions: ultraviolet (UV) irradiation for 24 h, high temperature and humidity (35°C, 85% relative humidity) for three days, and a rain-sheltered outdoor environment for seven days (with at least one sample per body fluid type under each condition). The results (Supplementary Fig. S8) indicated that in the outdoor exposure cohort, the PB, MB, and SA samples exhibited the most pronounced degradation, with two specific markers undetected in each sample. In the UV irradiation group, the SA, MB, and VS samples each had two undetected specific markers. The VS sample in the high-temperature and high-humidity cohort also had two undetected specific markers. Subsequently, the SVM-RFE multi-label classifier was employed to classify these 26 challenging aged samples, with the confusion matrix shown in Fig. 7D. The results demonstrated the robust predictive performance of the model, with only one PB sample and one SE sample misclassified in both the raw and prior-informed prediction outcomes, as the model predicted additional body fluid components alongside the true constituents.

In conclusion, despite the inevitable occurrence of a small number of marker drop-outs in degraded samples, the SVM-RFE multi-label classifier accurately classified most cases (EMR = 92.31%). These results further support the robust generalization performance of the model. Notably, while the panel maintains high BFID efficacy in challenging scenarios, marker drop-out poses a greater challenge for donor attribution in mixture deconvolution, which may lead to a significant decline in discriminatory power. Therefore, future optimization efforts should focus on identifying and incorporating additional markers with greater resistance to biological degradation.

### Forensic parameters and population genetic analysis

Initially, after LD analysis of all cMHs, pairs showing significant LD within the cMH set were merged into LD blocks (Supplementary Table S2-2). The swarm plot in Supplementary Fig. S9 illustrates the distributions of forensic parameters for the marker set after LD block integration. Specifically, the match probabilities and DP values ranged from 0.01 to 0.67 and 0.33 to 0.99, respectively, while the observed heterozygosity varied between 0.20 and 0.89. The polymorphism information content (PIC) and probability of exclusion values ranged from 0.17 to 0.91 and 0.03 to 0.77, respectively. Subsequently, the CDP for each body fluid was calculated, ranging from 0.99568126 for PB to 0.99999999 for VS. Furthermore, to investigate the heterogeneity of cMH allele frequencies across continental populations, we calculated the *F_ST_* values among the five continental populations based on the allele frequency data of all cMHs. The results revealed moderate genetic differentiation between the African populations and the other four continental populations, with particularly pronounced divergence among the African, European, and East Asian populations (Fig. 7E). Consequently, we performed PCA using the cMH genotype data of individuals from these three continental populations. The results demonstrated that the three populations formed distinct clusters with only minimal overlap (Fig. 7F). Collectively, these findings reveal significant allele frequency differentiation across distinct continental populations, highlighting the need for population-specific identification panels.

## Discussion

As the most common type of complex evidence in forensic practice, mixed stains typically involve multiple contributors, making the deconvolution of their components and donor attribution a persistent challenge in the BFID field. While the BFID method has increasingly matured in recent years [25, 51–53], the association fallacy during donor attribution remains a concern. To address this, Ingold et al. [3, 54] pioneered the concept of cSNPs and provided the first proof-of-concept for donor attribution using RNA profiling. Subsequently, various cSNP systems were developed on different technological platforms for dual-feature identification of body fluids [10, 11, 30, 46]. However, systems with limited polymorphism of markers show suboptimal attribution performance, whereas those with sufficient discriminatory power require numerous cSNPs, inevitably increasing analytical costs and multiplex primer design complexity. Compared to cSNPs, cMHs offer both short amplicons and high polymorphism, yet their potential in forensic BFID has not been systematically explored. Consequently, in this study, we developed a novel MPS-based cMH detection panel tailored for East Asian populations to substantially enhance donor attribution in body fluid stains.

To ensure the discriminatory power of the panel, 35 previously reported body fluid-specific genes were incorporated into this study. In addition, we identified six novel body fluid-specific genes that demonstrated significant specificity in their target body fluids. Specifically, *CA6* is predominantly expressed in salivary gland and mammary gland epithelial cells, as well as in ameloblasts. *CA6* encodes the secreted carbonic anhydrase VI, which plays critical roles in maintaining oral pH homeostasis, protecting teeth, and regulating taste perception [55, 56]. Previous studies have also indicated that the protein encoded by *CA6* exhibits remarkable SA specificity and can serve as a viable protein marker for SA identification [57]. The *ODAM* gene is primarily expressed in ameloblasts and encodes the odontogenic ameloblast-associated protein, which is essential for enamel formation and mineralization [58]. Furthermore, *ODAM* is significantly expressed in other oral tissues, including odontoblasts, salivary glands, and gingiva [59]. *COL4A2* is highly expressed in the brain, eyes, and muscles (particularly smooth muscle); the collagen type IV alpha 2 chain it encodes is a crucial component of basement membranes, where it is highly enriched, maintaining the structure and function of basement membrane [60]. *DKKL1* encodes a glycoprotein involved in the regulation of Wnt signaling; its elevated expression in testicular spermatids is closely associated with spermatogenesis and apoptosis regulation [61, 62]. *TUBA3E* is predominantly expressed across various developmental stages of spermatids and encodes tubulin alpha-3e, which participates in cytoskeletal assembly during meiosis of spermatocytes. Lastly, *LY9* is primarily expressed in T lymphocytes. It encodes the lymphocyte antigen 9 protein, a self-ligand receptor of the signaling lymphocytic activation molecule (SLAM) family, which participates in the regulation of innate and adaptive immune responses and in crosstalk between them by modulating the activation and differentiation of various immune cells.

This study demonstrates that incorporating cMH markers significantly enhances the panel's capacity for donor attribution in body fluid stains. Previously, a 35-cSNP panel and the enhanced "BFID-cSNP-6F" panel (46 cSNPs) developed by Ingold et al. and Hanson et al. yielded the theoretical CDP values of 0.7520–0.9996 and 0.9570–0.9970 across various body fluids, respectively [3, 7]. More recently, the targeted sequencing systems developed by Neis et al. [10] and Liu et al. [11], comprising 70 and 202 cSNPs, respectively, demonstrated CDP value ranges of 0.8025–0.9993 and 0.9972–0.9999, respectively. In comparison, the panel developed herein exhibited the CDP values of 0.99568126–0.99999999 across the five body fluid types. Given the limited number of reported body fluid-specific genes and the fact that the cumulative efficacy of multiplex systems is largely constrained by LD among markers, merging adjacent cSNPs into a cMH can effectively increase the PIC of a single marker. Consequently, the cMH-based approach achieves superior discriminatory power using fewer markers, thereby mitigating multiplex PCR design complexity and amplification competition. Furthermore, the existing systems are predominantly developed based on allele frequency data from global populations. However, allele frequency differentiation among populations often causes the actual utility of the panel to deviate from theoretical expectations. Therefore, developing a system specifically tailored for East Asian populations effectively improves its practical performance within this demographic. Notably, while systematic cross-continental comparisons are lacking, the previous studies have indicated that these genes exhibit robust body fluid-specific expression profiles across populations with diverse genetic backgrounds [3, 6, 10, 11, 23, 46]. This indirectly suggests that their expression levels are not significantly confounded by population genetic backgrounds. Among the 41 genes reported in this study, except for the six novel genes, the rest have been extensively validated across different populations. Consequently, although the donor attribution function of this panel is specific to East Asian populations, the machine learning classifiers constructed based on its gene expression data are expected to be broadly applicable to BFID across other continental populations. To ensure that RNA profiling is not confounded by residual gDNA, at least one primer in each pair should be designed to span an exon-exon junction. In this study, we initially prioritized this criterion when screening cMH markers to ensure the specificity of cDNA amplification. However, constrained by the sequence characteristics of certain transcripts, exon-exon junction-spanning primers could not be designed for some cMHs, introducing a potential risk of gDNA contamination. To address this, we employed an in-house optimized gDNA digestion protocol to treat total RNA extracts, ensuring that all sequencing reads originated exclusively from RNA transcripts. The efficacy of this protocol was validated by qPCR, which yielded no detectable gDNA amplification signals within 40 cycles for the treated samples (data not shown). This confirms the successful elimination of residual gDNA, thereby ensuring that cMH genotyping remains free from gDNA interference.

In practice, preparing comprehensive mixtures encompassing all possible component combinations and mixing ratios for model training poses a significant challenge. Although the machine learning classifier developed using large-scale simulated data demonstrated satisfactory performance, this strategy still presents certain limitations. Specifically, the simulated data are essentially proportional linear combinations of authentic single-source profiles. However, in actual casework, the total RNA yield from equal volumes of the same body fluid varies substantially among different individuals. Because RNA expression is confounded by factors such as degradation kinetics and microbial RNA abundance, it exhibits significant non-linear characteristics in real mixtures. Although we incorporated random feature dropout and Gaussian noise into the simulations to mimic amplicon loss and inter-individual biological variation, this strategy still cannot perfectly replicate the complex expression profiles of authentic mixtures. Additionally, because the simulated data only reflect the total read counts of each amplicon, they fail to capture complex allele read ratios, rendering them inapplicable to donor attribution tasks relying on allelic information. Thus, future studies should incorporate more genuine mixtures with diverse components and mixing ratios to construct large-scale datasets, helping to optimize the decision boundaries of the multi-label classifier and comprehensively evaluate panel robustness against scenarios such as severe allele drop-out. Moreover, avoiding the association fallacy during mixture deconvolution is crucial for accurate donor attribution. In binary mixtures, although slight allele balance shifts (<0.1) were observed for some cMHs, they rarely resulted in mixed genotypes, thereby enabling correct donor attribution. Notably, due to the complex physiological composition of MB, allele balance shifts manifest more complex patterns in MB–PB mixtures. When PB predominates, shifts in PB-specific cMHs are insignificant. Conversely, in highly imbalanced mixtures where MB is the major component, significant shifts in allele balance occur, leading to false homozygous or heterozygous calls in PB-specific cMHs, or allele drop-in that resulted in mixed genotypes. Therefore, future studies should include more highly unbalanced mixtures to optimize deconvolution strategies specifically tailored to these challenging samples.

In concordance analyses of RNA and DNA profiling, a small number of specific markers still yield false homozygosity or allelic imbalance [3, 10, 11]. These results are particularly pronounced in degraded samples and highly unbalanced mixtures. Consequently, future studies should elucidate the biological mechanisms underlying these aberrant genotypic profiles and establish standardized ATs and probabilistic interpretation frameworks based on large-scale cohort data. Such efforts will improve donor attribution accuracy in body fluid stains and enhance both the probative value and interpretability of these results as forensic evidence. Notably, although this study thoroughly evaluated the panel's deconvolution performance in highly unbalanced and complex mixtures, it has not yet addressed mixtures originating from related individuals. When contributors share a genetic relationship, the complexity of donor attribution increases substantially. The extensive sharing of genotypes among relatives exacerbates allele overlap and imposes stringent requirements on the polymorphism of the markers. Currently, the deconvolution of mixtures from related individuals remains thoroughly unexplored. However, given the critical practical value of addressing this challenge in resolving criminal cases involving family members, it is an indispensable step for the future translation of identification systems into forensic practice.

As a proof-of-concept, this study explored the feasibility of a dual-feature identification strategy for body fluid stains, integrating body fluid-specific cMH markers with machine learning algorithms and an *in silico* mixing strategy. The SVM-RFE multi-label classifier developed in this study achieved an EMR of ≥0.95 in the independent test and external validation sets. In contrast, a previous study by Roeder et al. using a scoring method yielded an average predictive accuracy of only 0.80 in five body fluids, with relatively low accuracy for MB and VS samples [63]. This indicated that incorporating machine learning algorithms can more accurately discern background read interference caused by cross-expression from true signals, significantly enhancing identification accuracy. However, the current study still presents certain limitations. The analyzed body fluid stains were exclusively collected from healthy, similarly aged volunteers, and prepared under controlled laboratory conditions, which cannot fully replicate the complex microenvironmental interference factors inherent in actual casework. Although the panel's robustness was validated on challenging aged samples, real forensic environments are far more complex and unpredictable; its efficacy on a broader array of authentic casework samples in such environments requires further evaluation. Additionally, the sample size was relatively limited and geographically restricted to Southern China. Specific gene expression, though highly correlated with body fluid types, also fluctuates with age, health, and lifestyle; thus, the current cohort and simulated datasets cannot encapsulate the full spectrum of inter-individual variations. Finally, while the models trained on the combined real and simulated dataset exhibited exceptional performance, their efficacy still requires further validation on a larger collection of authentic mixtures. Therefore, future studies should prioritize large-scale East Asian cohorts with broader age ranges and diverse subpopulations, as well as challenging samples recovered from various environments. This will improve the panel's robustness and generalizability, providing a population-representative foundation for applying this panel in forensic casework.

## Conclusion

This study developed a custom dual-purpose panel tailored for East Asian populations based on body fluid-specific mRNA cMHs, designed to improve BFID and donor attribution for complex mixed stains. We identified six novel body fluid-specific genes and systematically characterized their expression profiles in five body fluids. By integrating machine learning algorithms with an *in silico* mixing strategy, we developed a high-performance classifier capable of accurately inferring the tissue source of five forensically relevant body fluids, and fully explored the feasibility of integrating real and simulated data in forensic research. Furthermore, the panel demonstrated excellent donor attribution capability across these five fluid types, particularly for VS. Future research should incorporate additional body fluid-specific cMHs and integrate discriminative markers to distinguish target body fluids from potentially interfering tissues (e.g., skin, nasal mucosa, and rectal mucosa) to further improve the panel's efficiency and expand the scope of biological sample identification. As a proof-of-concept study, this work demonstrated the considerable potential of cMH markers to synergistically address both BFID and donor attribution analyses, thereby providing the crucial theoretical and experimental foundations for advancing the dual-feature identification of body fluid stains.

**Key Points**

- We developed a targeted mRNA-seq panel for dual-feature identification of body fluid stains, comprising 36 body fluid-specific genes containing 48 cMHs (encompassing 108 cSNPs), along with five genes lacking cMHs.
- The dual-purpose panel shows the promising potential for tissue source identification and donor attribution in body fluid stains.
- We developed a high-performance machine learning classifier capable of accurately identifying single-source body fluid stains and deconvoluting mixture components.
- The cumulative discrimination power of the panel ranges from 0.99568126 (peripheral blood) to 0.99999999 (vaginal secretion) in East Asian populations.

## Supplementary Material

Supplementary_material_bbag540

## Data and code availability statement

The raw sequencing data of single-source body fluid samples and the simulated data used for model construction are provided in Supplementary Table S8. The custom code utilized for simulated data generation, as well as for the construction and evaluation of the SVM-RFE multi-label classifier, has been deposited in a GitHub repository (https://github.com/lql19991211/SVM-RFE-model-for-BFID.git). Other original datasets used and/or analyzed during the current study are available from the corresponding author on reasonable request.

## Declaration of Large Language Model (LLM) Usage

During the preparation of this work, LLMs were utilized solely to assist with language refinement and code generation. The authors have meticulously reviewed all AI-assisted outputs and take full responsibility for the accuracy of the code and the content of the publication.
