# Supplementary material for: Leveraging mRNA cMH and machine learning for the deconvolution of mixed body fluid stains in East Asian populations
Source: Brief Bioinform. 2026 Oct 19;27(5):bbag540. doi: 10.1093/bib/bbag540 (PMC13679742; doi:10.1093/bib/bbag540)
Supplement: Supplementary file 1 [file supplementary_material_bbag540.zip › Supplementary Figure_bbag 504.docx]

**Supplementary Figure 1.** Relative expression (∆∆Ct) of six novel body fluid-specific genes identified in this study across diverse body fluids. ∆∆Ct is calculated as ∆Ct_target_ - ∆Ct_non-target_, where a value of 0 indicates that the fluid is the target body fluid for that gene. PB, SA, MB, VS, and SE represent peripheral blood, saliva, menstrual blood, vaginal secretion, and semen, respectively.

**Supplementary Figure 2.** Distribution of RNA sequencing read counts for amplicons exhibiting RNA/DNA genotypic discordance. Violin plots illustrating the distribution of RNA sequencing read counts for the cMH amplicons where profiling inconsistencies were observed. The internal box plots represent the median and interquartile ranges, with individual samples overlaid as scatter points. Black dots indicate samples with complete genotypic concordance between RNA and paired DNA sequencing, whereas red dots specifically highlight samples exhibiting discordant profiles (e.g., allele drop-out or severe allele imbalance). To maintain proportional consistency and facilitate visualization, the read counts for the SPTB-cMH2, TUBA3E-cMH, and CYP2A7-cMH3 amplicons were scaled down by a factor of 10 in the plot.

**Supplementary Figure 3**. Allele frequency distributions of cMHs in binary mixtures composed of the same body fluid type from two different donors. **A.** Peripheral blood –peripheral blood mixtures; **B.** Semen–semen mixtures; **C.** Saliva–Saliva mixtures. Alleles 1, 2, and 3 represent the respective alleles for each cMH.

**Supplementary Figure 4**. Genotyping results of body fluid-specific cMHs in complex mixtures consisting of three to five components. PB, SA, MB, VS, and SE represent peripheral blood, saliva, menstrual blood, vaginal secretion, and semen, respectively.

**Supplementary Figure 5.** Principal component analysis (PCA) of single-source body fluid samples across distinct sequencing batches.

**Supplementary Figure 6**. Probability-based decision inference process using the optimal decision thresholds of the SVM-RFE multi-label classifier on the independent test set. Red-highlighted probabilities represent the VS component predicted within each MB sample, ultimately classified as correct predictions post-masking. PB, SA, MB, VS, and SE represent peripheral blood, saliva, menstrual blood, vaginal secretion, and semen, respectively.

**Supplementary Figure 7**. Class-specific SHAP summary plots for the SVM-RFE multi-label classifier. The plots illustrate the top 15 key features with the highest contributions to model decisions across each body fluid type. **A.** Peripheral blood; **B.** Saliva; **C.** Menstrual blood; **D.** Vaginal secretion; and **E.** Semen. Features are ranked by their mean absolute SHAP values, and color coding represents relative feature expression levels.

**Supplementary Figure 8**. Detection status of all markers across 26 challenging aged samples. In the binary heatmap, dark purple represents successfully detected markers, while light purple indicates those that were not detected. The suffixes O, UV, and HTH in the sample names denote exposure to a rain-shielded outdoor environment for 7 days, ultraviolet irradiation for 24 hours, and high temperature and humidity (35°C, 85% relative humidity) for 3 days, respectively. PB, SA, MB, VS, and SE represent peripheral blood, saliva, menstrual blood, vaginal secretion, and semen, respectively.

**Supplementary Figure 9.** Beeswarm plot of the forensic parameters of the refined marker set comprising independent cMHs and merged LD blocks in the dual-purpose panel. Abbreviations: PIC, polymorphism information content; MP, match probability; DP, discrimination power; Hobs, observed heterozygosity; PE, probability of exclusion.
