# Supplementary material for: Leveraging mRNA cMH and machine learning for the deconvolution of mixed body fluid stains in East Asian populations
Source: Brief Bioinform. 2026 Oct 19;27(5):bbag540. doi: 10.1093/bib/bbag540 (PMC13679742; doi:10.1093/bib/bbag540)
Supplement: Supplementary file 1 [file supplementary_material_bbag540.zip › Supplementary Information_bbag 504.docx]

**Note 1**. Detailed information on the mixtures and compromised samples prepared in this study. Abbreviations: PB, peripheral blood; SA, saliva; MB, menstrual blood; VS, vaginal secretion; SE, semen.

**Table 1 Detailed information of the 40 mixtures**

| Mixtures | Components | Ratio I / RNA input amounts | Ratio II/RNA input amounts |
| --- | --- | --- | --- |
| Two components | VS–SE | 1:1/10:10 ng | 1:10/2:20 ng |
|  | VS–SA | 1:1/10:10 ng | 1:10/2:20 ng |
|  | SE–PB | 1:1/10:10 ng | 1:10/2:20 ng |
|  | SE– MB | 1:1/10:10 ng | 1:10/2:20 ng |
|  | MB–PB | 1:1/10:10 ng | 1:10/2:20 ng |
|  | MB–SA | 1:1/10:10 ng | 1:10/2:20 ng |
|  | SA–PB | 1:1/10:10 ng | 1:10/2:20 ng |
|  | SA–MB | 1:20/1:20 ng | – |
|  | SE–MB | 1:20/1:20 ng | – |
|  | PB–MB | 1:20/1:20 ng | – |
|  | SE–VS | 1:20/1:20 ng | – |
|  | PB–SA | 1:20/1:20 ng | – |
|  | VS–SA | 1:10/1:10 ng | 1:20/1:20 ng |
|  | SE–SA | 1:10/1:10 ng | 1:20/1:20 ng |
|  | MB–SA | 1:30/1:30 ng | – |
|  | MB–SE | 1:30/1:30 ng | – |
|  | MB–PB | 1:30/1:30 ng | – |
|  | VS–SE | 1:30/1:30 ng | – |
|  | SA–PB | 1:30/1:30 ng | – |
|  | SA–VS | 1:30/1:30 ng | – |
|  | SA–SE | 1:30/1:30 ng | – |
|  | SE–SE | 1:1/10:10 ng | – |
|  | SA–SA | 1:1/10:10 ng | – |
|  | PB–PB | 1:1/10:10 ng | – |
| Three components | SA–VS–SE | 1:1:1/10:10:10 ng | – |
|  | MB–VS–SE | 1:1:1/10:10:10 ng | – |
|  | MB–SE–PB | 1:1:1/10:10:10 ng | – |
|  | VS–SE–PB | 1:1:1/10:10:10 ng | – |
| Four components | SA–VS–SE–PB | 1:1:1:1/6:6:6:6 ng | – |
|  | VS–SE–PB–MB | 1:1:1:1/6:6:6:6 ng | – |
| Five components | SA–VS–SE–PB–MB | 1:1:1:1:1/5:5:5:5:5 ng | – |

**Table 2 Detailed information of the 26 compromised samples**

| Body fluid | Substrate–duration/Specific treatments | RNA input amounts |
| --- | --- | --- |
| PB | Gauze–38 days (ventilated indoor) | 25ng |
| PB | Swab–38 days (ventilated indoor) | 25ng |
| PB | Swab–346 days (ventilated indoor) | 25ng |
| SA | Facial tissue–3 days (ventilated indoor) | 25ng |
| SA | Facial tissue–200 days (ventilated indoor) | 25ng |
| MB | Swab–203 days (ventilated indoor) | 25ng |
| VS | Swab–97 days (ventilated indoor) | 25ng |
| SE | Condom–104 days (ventilated indoor) | 25ng |
| SE | Swab–2 years (ventilated indoor) | 25ng |
| PB/SA/VS/SE/MB | Outdoors, rain–sheltered (7 days) | 25ng |
| PB (*n*=2)/SA/VS/SE/MB | UV irradiation (24 h) | 25ng |
| PB (*n*=2)/SA/VS/SE/MB | High temperature and humidity (3 days; 35°C, 85% RH) | 25ng |

**Note 2**. Detailed workflow for screening candidate cSNPs in body fluid-specific genes from the dbSNP database and constructing cMHs:

(1) Target body fluid-specific genes were queried in the dbSNP database. The search type was strictly limited to "SNP" to retrieve all single nucleotide polymorphisms located within the candidate genes.

(2) To restrict the sequence scope exclusively to mature mRNA transcripts, the “Function Class” was filtered to include coding sequence variant, 3 Prime UTR variant, and 5 Prime UTR variant. Concurrently, the “Global MAF (minor allele frequency)” was restricted to a custom range of 0.1 to 0.5.

(3) The detailed characteristics of each remaining cSNP were individually evaluated. cSNPs exhibiting a MAF ≥ 0.1 within the East Asian populations of the 1000 Genomes Project were retained. Key metadata, including the gene symbol, chromosomal physical position, and MAF, were documented to establish the initial candidate cSNP set.

(4) Using NCBI reference sequence (RefSeq) accession numbers, mature mRNA transcript information for the corresponding genes was retrieved from the NCBI database, and the chromosomal physical positions of the exon-exon junctions were recorded.

(5) The aforementioned data were integrated into Microsoft Excel. The physical distances between adjacent cSNPs, as well as the distance from each cSNP to the nearest exon-exon junction, were calculated. Only adjacent cSNPs (two or more) located on the same exon with a physical distance of < 250 bp were retained. Furthermore, for these grouped cSNPs, the distance from the outermost cSNP to the nearest exon-exon junction was required to be ≤ 250 bp.

(6) Finally, the qualifying adjacent cSNPs obtained from the previous step were assembled to construct the cMH markers.

**Note 3**. The proportion of reads for each body fluid-specific amplicon across all non-target body fluid samples was calculated, with the 95th percentile of this distribution established as the threshold for determining the presence of the corresponding body fluid component in mixtures.

**Table 3 Non-specific expression read thresholds for mixture component characterization**

| Body fluid component | 95th percentile threshold (%) | Maximum background (%) | Negative samples (*N*) |
| --- | --- | --- | --- |
| Peripheral blood | 0.5 | 0.79 | 73 |
| Saliva | 1.5 | 2.71 | 75 |
| Menstrual blood | 0.8 | 3.19 | 98 |
| Vaginal secretion | 0.9 | 2.85 | 50 |
| Semen | 1.6 | 5.46 | 98 |

Note: Given the inherent weak non-specific expression between saliva- and vaginal secretion-specific genes, as well as the inevitable expression of peripheral blood and vaginal secretion-specific genes in menstrual blood, the scopes of non-target body fluids were adjusted during threshold calculation. Specifically, the non-target body fluids for saliva- and vaginal secretion-specific genes exclude each other, and those for vaginal secretion- and peripheral blood-specific genes exclude menstrual blood. This approach effectively mitigates the occurrence of false-negative results.

**Note 4**. Hyperparameter optimization and algorithm configuration details of the Random Forest (RF) and Support Vector Machine (SVM) multi-class models for inferring the tissue origin of single-source body fluid samples.

**Table 4 Hyperparameter search space and optimal configuration of the RF model**

| Hyperparameter | Description | Grid Search Space | Best Value |
| --- | --- | --- | --- |
| n_estimators | The number of trees in the forest | [10, 50, 100] | 50 |
| max_depth | The maximum depth of the tree | [None, 10, 20] | None (Unlimited) |
| max_features | The number of features to consider when looking for the best split | ['sqrt', 'log2'] | 'sqrt' |
| min_samples_split | The minimum number of samples required to split an internal node | [2, 5] | 2 |
| min_samples_leaf | The minimum number of samples required to be at a leaf node | [1, 2] | 1 |
| class_weight | Weights associated with classes (to handle class imbalance) | Fixed as 'balanced' | 'balanced' |
| random_state | Seed used by the random number generator (for reproducibility) | Fixed at a specific value | 42 |

**Table 5 Hyperparameter search space and optimal configuration of the SVM model**

| Hyperparameter | Description | Grid Search Space | Best Value |
| --- | --- | --- | --- |
| C | Regularization parameter (penalty parameter of the error term) | [0.1, 1, 10] | 1 |
| kernel | Specifies the kernel type to be used in the algorithm | ['linear', 'rbf', 'poly'] | 'linear' |
| gamma | Kernel coefficient (applicable for non-linear kernels) | ['scale', 'auto'] | 'scale' |
| probability | Whether to enable probability estimates | Fixed as True | True |
| class_weight | Weights associated with classes (to handle class imbalance) | Fixed as 'balanced' | 'balanced' |
| decision_function_shape | Multi-class classification strategy | Fixed as 'ovr' (One-vs-Rest) | 'ovr' |
| random_state | Seed used by the random number generator (for reproducibility) | Fixed at a specific value | 42 |

**Note 5**. This section details and compares the evaluation metrics and their 95% confidence intervals for various multi-label classification models across the independent test and the external validation sets. The comparison encompasses both the raw classification performance (i.e., without the masking procedure) and the prior-informed classification performance (i.e., with the masking adjustment). The results indicate that the XGBoost model exhibits the best raw classification performance. However, when evaluating the prior-informed classification performance, the SVM-RFE model slightly outperforms XGBoost and achieves the optimal performance. In forensic practice, classification performance that incorporates biological prior knowledge more authentically reflects a model's practical value in processing specific body fluid types (e.g., MB). The masking procedure effectively mitigates the "false positive" interferences caused by biological co-expression signatures, thereby significantly reducing the misclassification rate of MB component. Therefore, taking into account the overall performance and generalization stability of each model across both datasets, the SVM-RFE model is ultimately identified as the optimal model.

**Table 6 Comparison of raw and prior-informed classification performances across various multi-label classification models on the independent test and external validation sets**

| Models | Data set | Mask processing | Exact Match Ratio | Macro AUC | Hamming Loss | Macro Precision | Macro Recall | Macro F1-score |
| --- | --- | --- | --- | --- | --- | --- | --- | --- |
| SVM–RFE | Independent test set | No | 0.91  (0.84–1.00) | 1.00  (–) | 0.02  (0.00–0.03) | 0.94  (0.88–1.00) | 1.00  (–) | 0.97  (0.91–1.00) |
|  |  | Yes | 1.00  (–) | 1.00  (–) | 0.00  (0.00–0.00) | 1.00  (–) | 1.00  (–) | 1.00  (–) |
|  | External validation set | No | 0.83  (0.88–1.00) | 0.98  (0.96–1.00) | 0.04  (0.02–0.06) | 0.92  (0.88–0.97) | 1.00  (–) | 0.96  (0.93–0.98) |
|  |  | Yes | 0.95  (0.88–1.00) | 0.98  (0.96–1.00) | 0.01  (0.00–0.03) | 0.98  (0.94–1.00) | 1.00  (–) | 0.99  (0.97–1.00) |
| XGBoost | Independent test set | No | 1.00  (–) | 1.00  (–) | 0.00  (0.00–0.00) | 1.00  (–) | 1.00  (–) | 1.00  (–) |
|  |  | Yes | 1.00  (–) | 1.00  (–) | 0.00  (0.00–0.00) | 1.00  (–) | 1.00  (–) | 1.00  (–) |
|  | External validation set | No | 0.85  (0.73–0.95) | 0.99  (0.98–1.00) | 0.03  (0.01–0.06) | 0.97  (0.93–1.00) | 0.97  (0.92–1.00) | 0.96  (0.93–0.99) |
|  |  | Yes | 0.93  (0.83–1.00) | 0.99  (0.98–1.00) | 0.02  (0.00–0.04) | 1.00  (–) | 0.97  (0.93–1.00) | 0.98  (0.96–1.00) |
| RF | Independent test set | No | 0.95  (0.87–1.00) | 1.00  (–) | 0.01  (0.00–0.03) | 1.00  (–) | 0.95  (0.86–1.00) | 0.97  (0.91–1.00) |
|  |  | Yes | 0.95  (0.87–1.00) | 1.00  (–) | 0.01  (0.00–0.03) | 1.00  (–) | 0.95  (0.86–1.00) | 0.97  (0.91–1.00) |
|  | External validation set | No | 0.76  (0.75–0.80) | 0.99  (0.97–1.00) | 0.08  (0.05–0.11) | 0.98  (0.95–1.00) | 0.82  (0.75–0.88) | 0.88  (0.82–0.92) |
|  |  | Yes | 0.83  (0.78–0.88) | 0.99  (0.97–1.00) | 0.06  (0.05–0.08) | 1.00  (–) | 0.83  (0.76–0.89) | 0.91  (0.87–0.94) |
| Elastic Net LR | Independent test set | No | 0.84  (0.73–0.95) | 1.00  (–) | 0.05  (0.01–0.09) | 0.84  (0.72–0.95) | 1.00  (–) | 0.90  (0.81–0.97) |
|  |  | Yes | 0.92  (0.84–1.00) | 1.00  (–) | 0.03  (0.00–0.06) | 0.88  (0.76–1.00) | 1.00  (–) | 0.94  (0.85–1.00) |
|  | External validation set | No | 0.85  (0.75–0.95) | 0.99  (0.98–1.00) | 0.03  (0.01–0.05) | 0.93  (0.89–0.98) | 1.00  (–) | 0.96  (0.93–0.99) |
|  |  | Yes | 0.95  (0.88–1.00) | 0.99  (0.98–1.00) | 0.01  (0.00–0.03) | 0.98  (0.95–1.00) | 1.00  (–) | 0.99  (0.97–1.00) |
| KNN | Independent test set | No | 0.78  (0.65–0.92) | 1.00  (–) | 0.04  (0.02–0.07) | 0.86  (0.78–0.94) | 1.00  (–) | 0.92  (0.84–0.97) |
|  |  | Yes | 0.84  (0.74–0.95) | 1.00  (–) | 0.03  (0.01–0.05) | 0.89  (0.82–0.96) | 1.00  (–) | 0.94  (0.87–0.98) |
|  | External validation set | No | 0.63  (0.57–0.78) | 0.98  (0.95–1.00) | 0.10  (0.06–0.15) | 0.98  (0.94–1.00) | 0.80  (0.71–0.88) | 0.86  (0.78–0.92) |
|  |  | Yes | 0.73  (0.58–0.85) | 0.98  (0.95–1.00) | 0.07  (0.04–0.11) | 0.99  (0.96–1.00) | 0.85  (0.78–0.92) | 0.92  (0.87–0.96) |

Abbreviations: SVM-RFE, Support Vector Machine-Recursive Feature Elimination; XGBoost, eXtreme Gradient Boosting; RF, Random Forest; Elastic Net LR, Elastic Net Logistic Regression; KNN, K-Nearest Neighbors.

**Note 6**. Detailed results of pairwise McNemar's tests between three multi-label classification models, Support Vector Machine-Recursive Feature Elimination (SVM-RFE), Extreme Gradient Boosting (XGBoost), and Elastic Net Logistic Regression (Elastic Net LR), based on their prior-informed predictions on the independent test and external validation sets.

**Table 7 McNemar's test comparing the prior-informed classification performance of models on the independent test set**

| Model_A | Model_B | Both_Correct | A_Correct_B_Wrong | A_Wrong_B_Correct | Both_Wrong | *P*-value |
| --- | --- | --- | --- | --- | --- | --- |
| XGBoost | Elastic Net LR | 34 | 3 | 0 | 0 | 0.25 |
| SVM-RFE | Elastic Net LR | 34 | 3 | 0 | 0 | 0.25 |
| XGBoost | SVM | 37 | 0 | 0 | 0 | 1 |

Note: All *P* values were > 0.017 (Bonferroni-corrected threshold: 0.05/3), indicating no statistically significant difference in predictive efficacy between the two compared models.

**Table 8 McNemar's test comparing the prior-informed classification performance of models on the external validation set**

| Model_A | Model_B | Both_Correct | A_Correct_B_Wrong | A_Wrong_B_Correct | Both_Wrong | *P*-value |
| --- | --- | --- | --- | --- | --- | --- |
| XGBoost | Elastic Net LR | 36 | 1 | 2 | 1 | 1 |
| SVM-RFE | Elastic Net LR | 37 | 1 | 1 | 1 | 1 |
| XGBoost | SVM | 35 | 2 | 3 | 0 | 1 |

Note: All *P* values were > 0.017 (Bonferroni-corrected threshold: 0.05/3), indicating no statistically significant difference in predictive efficacy between the two compared models.

**Note 7.** Details of hyperparameter optimization and optimal algorithm configuration for the SVM-RFE multi-label classifier

**Table 9 Hyperparameter search space and optimal configuration of the SVM-RFE multi-label classifier**

| Pipeline Step | Hyperparameter | Description | Grid Search Space / Fixed Value | Best Value |
| --- | --- | --- | --- | --- |
| RFE (Feature Selection) | estimator | Base estimator for recursive feature elimination | Fixed as SVC | SVC |
|  | kernel | Kernel type for feature ranking | Fixed as 'linear' | 'linear' |
|  | step | Proportion of features removed per iteration | Fixed at 0.05 (5%) | 0.05 |
|  | n_features_to_select | Proportion of features retained | [0.3, 0.5, 0.8] | 0.3 |
| Final SVM (Classifier) | C | Regularization parameter | [0.1, 1, 10] | 10 |
|  | kernel | Kernel function type for final classification | ['linear', 'rbf'] | 'linear' |
|  | class_weight | Strategy to address class imbalance | Fixed as 'balanced' | 'balanced' |
|  | probability | Enables probability estimates for AUC calculation | Fixed as True | True |
| Global | random_state | Random seed for reproducibility | Fixed at 42 | 42 |

Note：The classifier utilizes a multioutput classifier framework, the RFE process was executed independently for each body fluid type. Consequently, while the optimal proportion of retained features is uniformly 0.3 (30%) across all classes, the specific combinations of features retained are distinct for each body fluid, achieving category-specific feature optimization.

**Note 8**. Detailed predictive performance evaluation of the reconstructed SVM-RFE multi-label classifier on the independent test and external validation sets following SHAP-based feature ablation.

**Table 10 Detailed performance metrics of the reconstructed SVM-RFE multi-label classifier after feature ablation**

| Data set | Exact Match Ratio | Macro AUC | Hamming Loss | Macro Precision | Macro Recall | Macro F1-score |
| --- | --- | --- | --- | --- | --- | --- |
| Independent test set | 0.95  (0.87–1.00) | 1.00  (–) | 0.01  (0.00–0.03) | 0.96  (0.90–1.00) | 1.00  (–) | 0.98  (0.93–1.00) |
| External validation set | 0.95  (0.88–1.00) | 0.99  (0.97–1.00) | 0.01  (0.00–0.03) | 0.98  (0.95–1.00) | 1.00  (–) | 0.99  (0.97–1.00) |
